# Eleven grand challenges in single-cell data science

**DOI:** 10.1186/s13059-020-1926-6

**Published:** 2020-02-07

**Authors:** David Lähnemann, Johannes Köster, Ewa Szczurek, Davis J. McCarthy, Stephanie C. Hicks, Mark D. Robinson, Catalina A. Vallejos, Kieran R. Campbell, Niko Beerenwinkel, Ahmed Mahfouz, Luca Pinello, Pavel Skums, Alexandros Stamatakis, Camille Stephan-Otto Attolini, Samuel Aparicio, Jasmijn Baaijens, Marleen Balvert, Buys de Barbanson, Antonio Cappuccio, Giacomo Corleone, Bas E. Dutilh, Maria Florescu, Victor Guryev, Rens Holmer, Katharina Jahn, Thamar Jessurun Lobo, Emma M. Keizer, Indu Khatri, Szymon M. Kielbasa, Jan O. Korbel, Alexey M. Kozlov, Tzu-Hao Kuo, Boudewijn P.F. Lelieveldt, Ion I. Mandoiu, John C. Marioni, Tobias Marschall, Felix Mölder, Amir Niknejad, Alicja Rączkowska, Marcel Reinders, Jeroen de Ridder, Antoine-Emmanuel Saliba, Antonios Somarakis, Oliver Stegle, Fabian J. Theis, Huan Yang, Alex Zelikovsky, Alice C. McHardy, Benjamin J. Raphael, Sohrab P. Shah, Alexander Schönhuth

**Affiliations:** 1 Algorithms for Reproducible Bioinformatics, Genome Informatics, Institute of Human Genetics, University Hospital Essen, University of Duisburg-Essen, Essen, Germany; 2 Department of Paediatric Oncology, Haematology and Immunology, Medical Faculty, Heinrich Heine University, University Hospital, Düsseldorf, Germany; 3 Computational Biology of Infection Research Group, Helmholtz Centre for Infection Research, Braunschweig, Germany; 4 Medical Oncology, Dana-Farber Cancer Institute, Harvard Medical School, Boston, USA; 5 Institute of Informatics, Faculty of Mathematics, Informatics and Mechanics, University of Warsaw, Warszawa, Poland; 6 Bioinformatics and Cellular Genomics, St Vincent’s Institute of Medical Research, Fitzroy, Australia; 7 Melbourne Integrative Genomics, School of BioSciences–School of Mathematics & Statistics, Faculty of Science, University of Melbourne, Melbourne, Australia; 8 Department of Biostatistics, Johns Hopkins University, Baltimore, MD USA; 9 Institute of Molecular Life Sciences and SIB Swiss Institute of Bioinformatics, University of Zürich, Zürich, Switzerland; 10 MRC Human Genetics Unit, Institute of Genetics and Molecular Medicine, University of Edinburgh, Western General Hospital, Edinburgh, UK; 11 The Alan Turing Institute, British Library, London, UK; 12 Department of Statistics, University of British Columbia, Vancouver, Canada; 13 Department of Molecular Oncology, BC Cancer Agency, Vancouver, Canada; 14 Data Science Institute, University of British Columbia, Vancouver, Canada; 15 Department of Biosystems Science and Engineering, ETH Zurich, Basel, Switzerland; 16 SIB Swiss Institute of Bioinformatics, Lausanne, Switzerland; 17 Leiden Computational Biology Center, Leiden University Medical Center, Leiden, The Netherlands; 18 Delft Bioinformatics Lab, Faculty of Electrical Engineering, Mathematics and Computer Science, Delft University of Technology, Delft, The Netherlands; 19 Molecular Pathology Unit and Center for Cancer Research, Massachusetts General Hospital Research Institute, Charlestown, USA; 20 Department of Pathology, Harvard Medical School, Boston, USA; 21 Broad Institute of Harvard and MIT, Cambridge, MA USA; 22 Department of Computer Science, Georgia State University, Atlanta, USA; 23 Computational Molecular Evolution Group, Heidelberg Institute for Theoretical Studies, Heidelberg, Germany; 24 Institute for Theoretical Informatics, Karlsruhe Institute of Technology, Karlsruhe, Germany; 25 Institute for Research in Biomedicine, The Barcelona Institute of Science and Technology, Barcelona, Spain; 26 Department of Pathology and Laboratory Medicine, University of British Columbia, Vancouver, Canada; 27 Life Sciences and Health, Centrum Wiskunde & Informatica, Amsterdam, The Netherlands; 28 Theoretical Biology and Bioinformatics, Science for Life, Utrecht University, Utrecht, The Netherlands; 29 Center for Molecular Medicine, University Medical Center Utrecht, Utrecht, The Netherlands; 30 Oncode Institute, Utrecht, The Netherlands; 31 Quantitative biology, Hubrecht Institute, Utrecht, The Netherlands; 32 Institute for Advanced Study, University of Amsterdam, Amsterdam, The Netherlands; 33 Department of Surgery and Cancer, The Imperial Centre for Translational and Experimental Medicine, Imperial College London, London, UK; 34 Centre for Molecular and Biomolecular Informatics, Radboud University Medical Center, Nijmegen, The Netherlands; 35 European Research Institute for the Biology of Ageing, University Medical Center Groningen, University of Groningen, Groningen, The Netherlands; 36 Bioinformatics Group, Wageningen University, Wageningen, The Netherlands; 37 Biometris, Wageningen University & Research, Wageningen, The Netherlands; 38 Department of Immunohematology and Blood Transfusion, Leiden University Medical Center, Leiden, The Netherlands; 39 Department of Biomedical Data Sciences, Leiden University Medical Center, Leiden, The Netherlands; 40 Genome Biology Unit, European Molecular Biology Laboratory, Heidelberg, Germany; 41 PRB lab, Delft University of Technology, Delft, The Netherlands; 42 Division of Image Processing, Department of Radiology, Leiden University Medical Center, Leiden, The Netherlands; 43 Computer Science & Engineering Department, University of Connecticut, Storrs, USA; 44 Cancer Research UK Cambridge Institute, Li Ka Shing Centre, University of Cambridge, Cambridge, UK; 45 Wellcome Trust Sanger Institute, Wellcome Genome Campus, Hinxton, UK; 46 European Molecular Biology Laboratory, European Bioinformatics Institute, Hinxton, UK; 47 Center for Bioinformatics, Saarland University, Saarbrücken, Germany; 48 Max Planck Institute for Informatics, Saarbrücken, Germany; 49 Institute of Pathology, University Hospital Essen, University of Duisburg-Essen, Essen, Germany; 50 Computation molecular design, Zuse Institute Berlin, Berlin, Germany; 51 Mathematics Department, Mount Saint Vincent, New York, USA; 52 Helmholtz Institute for RNA-based Infection Research, Helmholtz-Center for Infection Research, Würzburg, Germany; 53 Division of Computational Genomics and Systems Genetics, German Cancer Research Center–DKFZ, Heidelberg, Germany; 54 Institute of Computational Biology, Helmholtz Zentrum München–German Research Center for Environmental Health, Neuherberg, Germany; 55 Division of Drug Discovery and Safety, Leiden Academic Center for Drug Research–LACDR–Leiden University, Leiden, The Netherlands; 56 Department of Computer Science, Georgia State University, Atlanta, USA; 57 The Laboratory of Bioinformatics, I.M. Sechenov First Moscow State Medical University, Moscow, Russia; 58 Department of Computer Science, Princeton University, Princeton, USA; 59 Computational Oncology, Department of Epidemiology and Biostatistics, Memorial Sloan Kettering Cancer Center, New York, USA

## Abstract

The recent boom in microfluidics and combinatorial indexing strategies, combined with low sequencing costs, has empowered single-cell sequencing technology. Thousands—or even millions—of cells analyzed in a single experiment amount to a data revolution in single-cell biology and pose unique data science problems. Here, we outline eleven challenges that will be central to bringing this emerging field of single-cell data science forward. For each challenge, we highlight motivating research questions, review prior work, and formulate open problems. This compendium is for established researchers, newcomers, and students alike, highlighting interesting and rewarding problems for the coming years.

## Introduction

Since being highlighted as “Method of the Year” in 2013 [1], sequencing of the genetic material of individual cells has become routine when investigating cell-to-cell heterogeneity. Single-cell measurements of both RNA and DNA, and more recently also of epigenetic marks and protein levels, can stratify cells at the finest resolution possible.

Single-cell RNA sequencing (scRNA-seq) enables transcriptome-wide gene expression measurement at single-cell resolution, allowing for cell type clusters to be distinguished (for an early example, see [2]), the arrangement of populations of cells according to novel hierarchies, and the identification of cells transitioning between states. This can lead to a much clearer view of the dynamics of tissue and organism development, and on structures within cell populations that had so far been perceived as homogeneous. In a similar vein, analyses based on single-cell DNA sequencing (scDNA-seq) can highlight somatic clonal structures (e.g., in cancer, see [3, 4]), thus helping to track the formation of cell lineages and provide insight into evolutionary processes acting on somatic mutations.

The opportunities arising from single-cell sequencing (sc-seq) are enormous: only now is it possible to re-evaluate hypotheses about differences between pre-defined sample groups at the single-cell level—no matter if such sample groups are disease subtypes, treatment groups, or simply morphologically distinct cell types. It is therefore no surprise that enthusiasm about the possibility to screen the genetic material of the basic units of life has continued to grow. A prominent example is the Human Cell Atlas [5], an initiative aiming to map the numerous cell types and states comprising a human being.

Encouraged by the great potential of investigating DNA and RNA at the single-cell level, the development of the corresponding experimental technologies has experienced considerable growth. In particular, the emergence of microfluidics techniques and combinatorial indexing strategies [6–10] has led to hundreds of thousands of cells routinely being sequenced in one experiment. This development has even enabled a recent publication analyzing millions of cells at once [11]. Sc-seq datasets comprising very large cell numbers are becoming available worldwide, constituting a data revolution for the field of single-cell analysis.

These vast quantities of data and the research hypotheses that motivate them need to be handled in a computationally efficient and statistically sound manner [12]. As these aspects clearly match a recent definition of “Data Science” [13], we posit that we have entered the era of single-cell data science (SCDS).

SCDS exacerbates many of the data science issues arising in bulk sequencing, but it also constitutes a set of new, unique challenges for the SCDS community to tackle. Limited amounts of material available per cell lead to high levels of uncertainty about observations. When amplification is used to generate more material, technical noise is added to the resulting data. Further, any increase in resolution results in another—rapidly growing—dimension in data matrices, calling for scalable data analysis models and methods. Finally, no matter how varied the challenges are—by research goal, tissue analyzed, experimental setup, or just by whether DNA or RNA is sequenced—they are all rooted in data science, i.e., are computational or statistical in nature. Here, we propose the data science challenges that we believe to be among the most relevant for bringing SCDS forward.

This catalog of SCDS challenges aims at focusing the development of data analysis methods and the directions of research in this rapidly evolving field. It shall serve as a compendium for researchers of various communities, looking for rewarding problems that match their personal expertise and interests. To make it accessible to these different communities, we categorize challenges into the following: transcriptomics (see “Challenges in single-cell transcriptomics”), genomics (see the “Challenges in single-cell genomics”), and phylogenomics (see “Challenges in single-cell phylogenomics”). For each challenge, we provide a thorough review of the status relative to existing approaches and point to possible directions of research to solve it.

Several themes and aspects recur across the boundaries of research communities and methodological approaches. We represent these overlaps in three different ways. First, we decided to discuss some problems in multiple contexts, highlighting the relevant aspects for the respective research communities (e.g., data sparsity in transcriptomics and genomics). Second, we separately introduce recurring themes (see “Single-cell data science: recurring themes”), thereby keeping respective discussions in each challenge succinct. Third, if challenges were identified as independent of the chosen categorization, they are discussed as recapitulatory challenges at the end (see “Overarching challenges”).

## Single-cell data science: recurring themes

A number of challenging themes are common to many or all single-cell analyses, regardless of the particular assay or data modality generated. We will start our review by introducing them. Later, when discussing the specific challenges, we will refer to these broader themes wherever appropriate and outline what they mean in the particular context. If challenges covered in later sections are particularly entangled with the broader themes listed here, we will also refer to them from within this section.

The themes may reflect issues one also experiences when analyzing bulk sequencing data. However, even if not unique to single-cell experiments, these issues may dominate the analysis of sc-seq data and therefore require particular attention. The two most urgent elementary themes, not necessarily unique to sc-seq, are the need to quantify measurement uncertainty (see “Quantifying uncertainty of measurements and analysis results”) and the need to benchmark methods systematically, in a way that highlights the metrics that are particularly critical in sc-seq. Since the latter is of central importance and an aspect that has gained visibility only recently, we not only mention its importance in relevant challenges, but also consider it a challenge in its own right (see “Challenge XI: Validating and benchmarking analysis tools for single-cell measurements”).

We identify three sweeping themes that are more specific to sc-seq, exacerbated by the rapid advances in experimental technologies. First, there is a need to scale to higher dimensional data, be it more cells measured or more data measured per cell (see “Scaling to higher dimensionalities: more cells, more features, and broader coverage”). This need often arises in combination with a second one: the need to integrate data across different types of single-cell measurements (e.g., RNA, DNA, proteins, and methylation) and across samples, be it from different time points, treatment groups, or even organisms. This integration theme runs throughout multiple challenges and is so central that we consider it a challenge worth highlighting (see “Challenge X: Integration of single-cell data across samples, experiments, and types of measurement”). Third, the possibility to operate on the finest levels of resolution casts an important, overarching question: what level of resolution is appropriate relative to the particular research question one has in mind (see “Varying levels of resolution”)? We will start by qualifying this last one.

### Varying levels of resolution

Sc-seq allows for a fine-grained definition of cell types and states. Hence, it allows for characterizations of cell populations that are significantly more detailed than those supported by bulk sequencing experiments. However, even though sc-seq operates at the most basic level, mapping cell types and states at a particular level of resolution of interest may be challenging: Achieving the targeted level of resolution or granularity for the intended map of cells may require substantial methodological efforts and will depend on whether the research question allows for a certain freedom in terms of resolution and on the limits imposed by the particular experimental setup.

When drawing maps of cell types and states, it is important that they (i) have a structure that recapitulates both tissue development and tissue organization; (ii) account for continuous cell states in addition to discrete cell types (i.e., reflecting cell state trajectories within cell types and smooth transitions between cell types, as observed in tissue generation); (iii) allow for choosing the level of resolution flexibly (i.e., the map should possibly support zoom-type operations, to let the researcher choose the desired level of granularity with respect to cell types and states conveniently, ranging from whole organisms via tissues to cell populations and cellular subtypes); and (iv) include biological and functional annotation wherever available and helpful in the intended functional context.

**Fig. 1:**
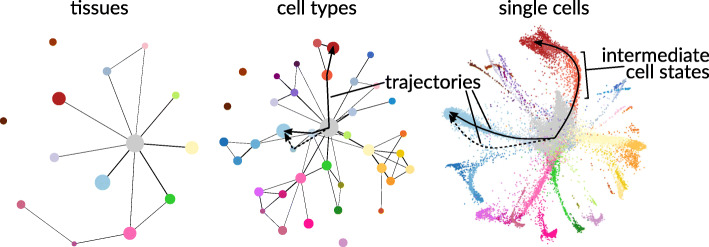
Different levels of resolution are of interest, depending on the research question and the data available. Thus, analysis tools and reference systems (such as cell atlases) will have to accommodate multiple levels of resolution from whole organs and tissues over discrete cell types to continuously mappable intermediate cell states, which are indistinguishable even at the microscopic level. A graph abstraction that enables such multiple levels of focus is provided by PAGA [14], a structure that allows for discretely grouping cells, as well as inferring trajectories as paths through a graph

An exemplary illustration of how maps of cell types and states can support different levels of resolution is the structure-rich topologies generated by PAGA based on scRNA-seq [14], see Fig. 1,^1^. At the highest levels of resolution, these topologies also reflect intermediate cell states and the developmental trajectories passing through them. A similar approach that also allows for consistently zooming into more detailed levels of resolution is provided by hierarchical stochastic neighbor embedding (HSNE, Pezzotti et al. [15]), a method pioneered on mass cytometry datasets [16, 17]. In addition, manifold learning [18, 19] and metric learning [20, 21] may provide further theoretical support for even more accurate maps, because they provide sound theories about reasonable, continuous distance metrics, instead of just distinct, discrete clusters.

### Quantifying uncertainty of measurements and analysis results

The amount of material sampled from single cells is considerably less than that used in bulk experiments. Signals become more stable when individual signals are summarized (such as in a bulk experiment); thus, the increase in resolution due to sc-seq also means a reduction of the stability of the supporting signals. The reduction in signal stability, in turn, implies that data becomes substantially more uncertain and tasks so far considered routine, such as single nucleotide variation (SNV) calling in bulk sequencing, require considerable methodological care with sc-seq data.

These issues with data quality and in particular missing data pose challenges that are unique to sc-seq, and are thus at the core of several challenges: regarding scDNA-seq data quality (see “Challenges in single-cell genomics”) and especially regarding missing data in scDNA-seq (“Challenge VI: Dealing with errors and missing data in the identification of variation from single-cell DNA sequencing data”) and scRNA-seq (“Challenge I: Handling sparsity in single-cell RNA sequencing”). In contrast, the non-negligible batch effects that scRNA-seq can suffer from reflect a common problem in high-throughput data analysis [22], and thus are not discussed here (although in certain protocols such effects can be alleviated by careful use of negative control data in the form of spike-in RNA of known content and concentration, see, for example, BEARscc [23]).

Optimally, sc-seq analysis tools would accurately quantify all uncertainties arising from experimental errors and biases. Such tools would prevent the uncertainties from propagating to the intended downstream analyses in an uncontrolled manner, and rather translate them into statistically sound and accurately quantified qualifiers of final results.

### Scaling to higher dimensionalities: more cells, more features, and broader coverage

The current blossoming of experimental methods poses considerable statistical challenges, and would do so even if measurements were not affected by errors and biases. The increase in the number of single cells analyzed per experiment translates into more data points being generated, requiring methods to scale rapidly. Some scRNA-seq SCDS methodology has started to address scalability [12, 24–27], but the respective issues have not been fully resolved and experimental methodology will scale further. For scDNA-seq, experimental methodology has just been scaling up to more cells recently (see Table 1 and “Challenge VII: Scaling phylogenetic models to many cells and many sites”), making this a pressing challenge in the development of data analysis methods.

**Table 1:** Whole genome amplification: recent improvements

Recent improvements of whole genome amplification (WGA) methods promise to reduce amplification biases and errors, while scaling throughput to larger cell numbers:
1. Improved coverage uniformity for multiple displacement amplification (MDA) has been achieved using droplet microfluidics-based methods (eWGA [28]; sd-MDA [29]; ddMDA [30]). A second approach has been to couple the *Φ*29 DNA polymerase to a primase to reduce priming bias [31].
2. One way to reduce the amplification error rate of the polymerase chain reaction (PCR)-based methods (including multiple annealing and looping-based amplification cycles (MALBAC)) would be to employ a thermostable polymerase (necessary for use in PCR) with proof-reading activity similar to *Φ*29 DNA polymerase, but we are not aware of any PCR DNA polymerases with a fidelity in the range of *Φ*29 DNA polymerase [32].
3. Three newer methods use an entirely different approach: they randomly insert transposons into the whole genome and then leverage these as priming sites for amplification and library preparation. Transposon Barcoded (TnBC) library preparation (with a PCR amplification, [33]) and direct library preparation (DLP) (with a shallow library without any amplification, [34]) allow only for copy number variation (CNV) calling, but DLP scales up to 80,000 single cells [35]. Linear—as opposed to exponential—Amplification via Transposon Insertion (LIANTI, [36]) also addresses amplification errors: all copies are generated based on the original genomic DNA through in vitro transcription. With errors unique to individual barcoded copies, the authors report a false positive rate that is even lower than for MDA [36].

Beyond basic scRNA-seq and scDNA-seq experiments, various assays have been proposed to measure chromatin accessibility [37, 38], DNA methylation [39], protein levels [40], protein binding, and also for performing multiple simultaneous measurements [41, 42] in single cells. The corresponding increase in experimental choices means another possible inflation of feature spaces.

In parallel to the increase in the number of cells queried and the number of different assays possible, the increase of the resolution per cell of specific measurement types causes a steady increase of the dimensionality of corresponding data spaces. For the field of SCDS, this amounts to a severe and recurring case of the “curse of dimensionality” for all types of measurements. Here again, scRNA-seq-based methods are in the lead when trying to deal with feature dimensionality, while scDNA-seq-based methodology (which includes epigenome assays) has yet to catch up.

Finally, there are efforts to measure multiple feature types in parallel, e.g., from scDNA-seq (see “Challenge VIII: Integrating multiple types of variation into phylogenetic models”). Also, with spatial and temporal sampling becoming available (see “Challenge V: Finding patterns in spatially resolved measurements” and “Challenge IX: Inferring population genetic parameters of tumor heterogeneity by model integration”), data integration methods need to scale to more and new types of context information for individual cells (see “Challenge X: Integration of single-cell data across samples, experiments, and types of measurement” for a comprehensive discussion of data integration approaches).

## Challenges in single-cell transcriptomics

### Challenge I: Handling sparsity in single-cell RNA sequencing

A comprehensive characterization of the transcriptional status of individual cells enables us to gain full insight into the interplay of transcripts within single cells. However, scRNA-seq measurements typically suffer from large fractions of observed zeros, where a given gene in a given cell has no unique molecular identifiers or reads mapping to it. The term “dropout” is often used to denote observed zero values in scRNA-seq data. But this term usually conflates two distinct types of zero values: those attributable to methodological noise, where a gene is expressed but not detected by the sequencing technology, and those attributable to biologically-true absence of expression. Thus, we recommend against the term “dropout” as a catch-all term for observed zeros. Beyond biological variation in the number of unexpressed genes, the proportion of observed zeros, or degree of sparsity, is attributed to technical limitations [43, 44]. Those can result in artificial zeros that are either systematic (e.g., sequence-specific mRNA degradation during cell lysis) or that occur by chance (e.g., barely expressed transcripts that—at the same expression level, due to sampling variation—will sometimes be detected and sometimes not). Accordingly, the degree of sparsity depends on the scRNA-seq platform used, the sequencing depth, and the underlying expression level of the gene.

**Fig. 2:**
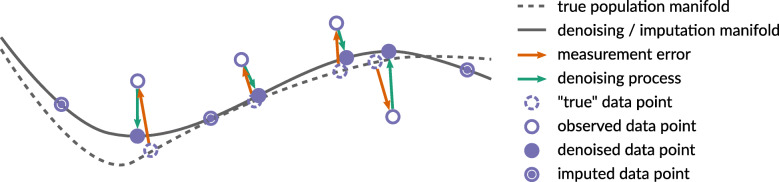
Measurement error requires denoising methods or approaches that quantify uncertainty and propagate it down analysis pipelines. Where methods cannot deal with abundant missing values, imputation approaches may be useful. While the true population manifold that generated data is never known, one can usually obtain some estimation of it that can be used for both denoising and imputation

Sparsity in scRNA-seq data can hinder downstream analyses and is still challenging to model or handle appropriately, calling for further method development. Sparsity pervades all aspects of scRNA-seq data analysis, but in this challenge, we focus on the linked problems of learning latent spaces and “imputing” expression values from scRNA-seq data (Fig. 2). Imputation approaches are closely linked to the challenges of normalization. But whereas normalization generally aims to make expression values between cells and experiments more comparable to each other, imputation approaches aim to achieve adjusted data values that better represent the true expression values. Imputation methods could therefore be used for normalization, but do not entail all possible or useful approaches to normalization.

**Fig. 3:**
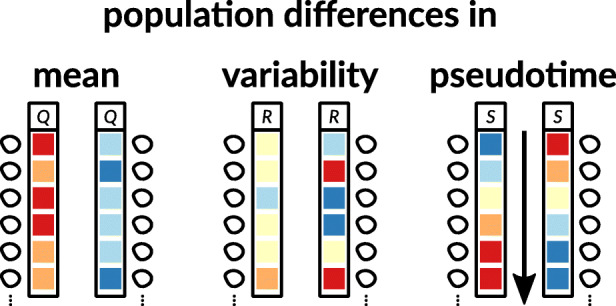
Differential expression of a gene or transcript between cell populations. The top row labels the specific gene or transcript, as is also done in Fig. 6. A difference in *mean* gene expression manifests in a consistent difference of gene expression across all cells of a population (e.g., high vs. low). A difference in *variability* of gene expression means that in one population, all cells have a very similar expression level, whereas in another population, some cells have a much higher expression and some a much lower expression. The resulting average expression level may be the same, and in such cases, only single-cell measurements can find the difference between populations. A difference *across pseudotime* is a change of expression within a population, for example, along a developmental trajectory (compare Fig. 1). This also constitutes a difference between cell populations that is not apparent from population averages, but requires a pseudo-temporal ordering of measurements on single cells

#### Status

The imputation of missing values has been very successful for genotype data [45]. Crucially, when imputing genotypes, we typically know which data are missing (e.g., when no genotype call is possible due to no coverage of a locus; although see the “Challenge VI: Dealing with errors and missing data in the identification of variation from single-cell DNA sequencing data” for the challenges with scDNA-seq data). In addition, rich sources of external information are available (e.g., haplotype reference panels). Thus, genotype imputation is now highly accurate and a commonly used step in data processing for genetic association studies [46].

The situation is somewhat different for scRNA-seq data, as we do not routinely have external reference information to apply (see “Challenge III: Mapping single cells to a reference atlas”). In addition, we can never be sure which of the observed zeros represent “missing data” and which accurately represent a true absence of gene expression in the cell [43].

In general, two broad approaches can be applied to tackle this problem of sparsity: (i) use statistical models that inherently model the sparsity, sampling variation, and noise modes of scRNA-seq data with an appropriate data generative model (i.e., quantifying uncertainty, see the “Quantifying uncertainty of measurements and analysis results”), or (ii) attempt to “impute” values for observed zeros (ideally the technical zeros; sometimes also non-zero values) that better approximate the true gene expression levels (Fig. 2). We prefer to use the first option where possible, and for many single-cell data analysis problems, there already are statistical models appropriate for sparse count data that should be used or extended (e.g., for differential expression analysis, see the “Challenge II: Defining flexible statistical frameworks for discovering complex differential patterns in gene expression”). However, there are many cases where the appropriate models are not available and accurate imputation of technical zeros would allow better results from downstream methods and algorithms that cannot handle sparse count data. For example, depending on the amount of sparsity, imputation could potentially improve results of dimension reduction, visualization, and clustering applications.

We define three broad (and often overlapping) categories of methods that can be used to “impute” scRNA-seq data in the absence of an external reference (Table 2): (A) *Model-based imputation methods* of technical zeros use probabilistic models to identify which observed zeros represent technical rather than biological zeros. They aim to impute expression levels only for the technical zeros, leaving other observed expression levels untouched. (B) *Data-smoothing methods* define a “similarity” between cells (e.g., cells that are neighbors in a graph or occupy a small region in a latent space) and adjust expression values for each cell based on expression values in similar cells. These methods usually adjust all expression values, including technical zeros, biological zeros, and observed non-zero values. (C) *Data-reconstruction methods* typically aim to define a latent space representation of the cells. This is often done through matrix factorization (e.g., principal component analysis) or, increasingly, through machine learning approaches (e.g., variational autoencoders that exploit deep neural networks to capture non-linear relationships). Both matrix factorization methods and autoencoders (among others) are able to “reconstruct” the observed data matrix from low-rank or simplified representations. The reconstructed data matrix will typically no longer be sparse (with many zeros), and the implicitly “imputed” data (or estimated latent spaces if using, for example, variational autoencoders) can be used for downstream applications such as clustering or trajectory inference (see “Challenge IV: Generalizing trajectory inference”). A fourth—distinct—category is (T) imputation with an external dataset or reference, using it for transfer learning.

**Table 2:** Short description of methods for the imputation of missing data in scRNA-seq data

A: model-based imputation		
bayNorm	Binomial model, empirical Bayes prior	[47]
BISCUIT	Gaussian model of log counts, cell- and cluster-specific parameters	[48]
CIDR	Decreasing logistic model (DO), non-linear least-squares regression (imp)	[49]
SAVER	NB model, Poisson LASSO regression prior	[50]
ScImpute	Mixture model (DO), non-negative least squares regression (imp)	[51]
scRecover	ZINB model (DO identification only)	[52]
VIPER	Sparse non-negative regression model	[53]
B: data smoothing		
DrImpute	*k*-means clustering of PCs of correlation matrix	[54]
knn-smooth	*k*-nearest neighbor smoothing	[55]
LSImpute	Locality sensitive imputation	[56]
MAGIC	Diffusion across nearest neighbor graph	[57]
netSmooth	Diffusion across PPI network	[58]
C: data reconstruction, matrix factorization		
ALRA	SVD with adaptive thresholding	[59]
ENHANCE	Denoising PCA with aggregation step	[60]
scRMD	Robust matrix decomposition	[61]
consensus NMF	Meta-analysis approach to NMF	[62]
f-scLVM	Sparse Bayesian latent variable model	[63]
GPLVM	Gaussian process latent variable model	[64]
pCMF	Probab. count matrix factorization with Poisson model	[65]
scCoGAPS	Extension of NMF	[66]
SDA	Sparse decomposition of arrays (Bayesian)	[67]
ZIFA	ZI factor analysis	[68]
ZINB-WaVE	ZINB factor model	[69]
C: data reconstruction, machine learning		
AutoImpute	AE, no error back-propagation for zero counts	[70]
BERMUDA	AE for cluster batch correction (MMD and MSE loss function)	[71]
DeepImpute	AE, parallelized on gene subsets	[72]
DCA	Deep count AE (ZINB / NB model)	[73]
DUSC / DAWN	Denoising AE (PCA determines hidden layer size)	[74]
EnImpute	Ensemble learning consensus of other tools	[75]
Expression Saliency	AE (Poisson negative log-likelihood loss function)	[76]
LATE	Non-zero value AE (MSE loss function)	[77]
Lin_DAE	Denoising AE (imputation across *k*-nearest neighbor genes)	[78]
SAUCIE	AE (MMD loss function)	[79]
scScope	Iterative AE	[80]
scVAE	Gaussian-mixture VAE (NB / ZINB / ZIP model)	[81]
scVI	VAE (ZINB model)	[82]
scvis	VAE (objective function based on latent variable model and t-SNE)	[83]
VASC	VAE (denoising layer; ZI layer, double-exponential and Gumbel distribution)	[84]
Zhang_VAE	VAE (MMD loss function)	[85]
T: using external information		
ADImpute	Gene regulatory network information	[86]
netSmooth	PPI network information	[58]
SAVER-X	Transfer learning with atlas-type resources	[87]
SCRABBLE	Matched bulk RNA-seq data	[88]
TRANSLATE	Transfer learning with atlas-type resources	[77]
URSM	Matched bulk RNA-seq data	[89]

Imputation methods using only data from within a dataset are roughly categorized approaches A (model-based), B (data smoothing), and C (data reconstruction), with the latter further differentiated into matrix factorization and machine learning approaches. In contrast to these methods, those in category T (for transfer learning) also use information external to the dataset to be analyzed

*AE* autoencoder, *DO* dropout, *imp* imputation, *MMD* maximum mean discrepancy, *MSE* mean squared error, *NB* negative binomial, *NMF* non-negative matrix factorization, *P* Poisson, *PC* principal component, *PCA* principal component analysis, *PPI* protein-protein interaction, *SVD* singular value decomposition, *VAE* variational autoencoder, *ZI* zero-inflated

The first category of methods generally seeks to infer a probabilistic model that captures the data generation mechanism. Such generative models can be used to probabilistically determine which observed zeros correspond to technical zeros (to be imputed) and which correspond to biological zeros (to be left alone). There are many model-based imputation methods already available that use ideas from clustering (e.g., *k*-means), dimension reduction, regression, and other techniques to impute technical zeros, oftentimes combining ideas from several of these approaches (Table 2 (A)).

Data-smoothing methods adjust all gene expression levels based on expression levels in “similar” cells, aiming to “denoise” the values (Fig. 2). Several such methods have been proposed to handle imputation problems (Table 2 (B)). To take a simplified example (Fig. 2), we might imagine that single cells originally refer to points along a curve across a two-dimensional space. Projecting data points onto that curve eventually allows imputation of the “missing” values (but all points are adjusted, or smoothed, not just true technical zeros).

A major task in the analysis of high-dimensional single-cell data is to find low-dimensional representations of the data that capture the salient biological signals and render the data more interpretable and amenable to further analyses. As it happens, the matrix factorization and latent-space learning methods used for that task also provide a third route for imputation: they can *reconstruct* the observed data matrix from simplified representations of it.

Principal component analysis (PCA) is one standard matrix factorization method that can be applied to scRNA-seq data (preferably after suitable data normalization) as are other widely used general statistical methods like independent component analysis (ICA) and non-negative matrix factorization (NMF). As (linear) matrix factorization methods, PCA, ICA, and NMF decompose the observed data matrix into a “small” number of factors in two low-rank matrices, one representing cell-by-factor weights and one gene-by-factor loadings. Many matrix factorization methods with tweaks for single-cell data have been proposed in recent years (Table 2 (C)), with some specifically intended for imputation (ALRA, ENHANCE, scRMD).

Additionally, machine learning methods have been proposed for scRNA-seq data analysis that can, but need not, use probabilistic data generative processes to capture low-dimensional or latent space representations of a dataset (Table 2 (C)). Some of them are expressly aimed at imputation (e.g., AutoImpute, DeepImpute, EnImpute, DCA, and scVI). But even if imputation is not the main focus, such methods can generate “imputed” expression values as an upshot of a model primarily focused on other tasks, like learning latent spaces, clustering, batch correction, or visualization (and often several of these tasks simultaneously).

Finally, a small number of scRNA-seq imputation methods extend approaches from any (combination) of the three categories above by incorporating information external to the current dataset (Table 2 (T)). Approaches using cell atlas-type reference resources are further discussed in the “Challenge III: Mapping single cells to a reference atlas” section and classified as approach +X+S in the “Challenge X: Integration of single-cell data across samples, experiments, and types of measurement” (see Fig. 6 and Table 4).

#### Open problems

A major challenge in this context is the circularity that arises when imputation solely relies on information that is internal to the imputed dataset. This circularity can artificially amplify the signal contained in the data, leading to inflated correlations between genes or cells. In turn, this can introduce false positives in downstream analyses such as differential expression testing and gene network inference [90]. Handling batch effects and potential confounders requires further work to ensure that imputation methods do not mistake unwanted variation from technical sources for biological signal. In a similar vein, single-cell experiments are affected by various uncertainties (see “Quantifying uncertainty of measurements and analysis results”). Approaches that allow quantification and propagation of the uncertainties associated with expression measurements (see “Quantifying uncertainty of measurements and analysis results”) may help to avoid problems associated with “overimputation” and the introduction of spurious signals noted by Andrews and Hemberg [90].

To avoid this circularity, it is important to identify reliable external sources of information that can inform the imputation process. One possibility is to exploit external reference panels (like in the context of genetic association studies). Such panels are not generally available for scRNA-seq data, but ongoing efforts to develop large scale cell atlases (e.g., [5]) could provide a valuable resource for this purpose. Some methods have been extended to allow the use of such resources (e.g., SAVER-X and TRANSLATE), but this will need to be done for all approaches (see “Challenge III: Mapping single cells to a reference atlas”).

A second approach to avoid circularity is the systematic integration of known biological network structures in the imputation process. This can be achieved by encoding network structure knowledge as prior information, as proposed by ADImpute and netSmooth and the tool by Lin et al. [78].

Finally, a third way of avoiding circularity in imputation is to explore complementary types of data that can inform scRNA-seq imputation. This idea was adopted in SCRABBLE and URSM, where an external reference is defined by bulk expression measurements from the same population of cells for which imputation is performed. Of course, such orthogonal information can also be provided by different types of molecular measurements (see “Challenge X: Integration of single-cell data across samples, experiments, and types of measurement”). Methods designed to integrate multi-omics data could then be extended to enable scRNA-seq imputation, for example, through generative models that explicitly link scRNA-seq with other data types (e.g., clonealign [91]) or by inferring a shared low-dimensional latent structure (e.g., MOFA [92]) that could be used within a data-reconstruction framework.

With the proliferation of alternative methods, comprehensive benchmarking is urgently required—as for all areas of single-cell data analysis (see “Challenge XI: Validating and benchmarking analysis tools for single-cell measurements”). Early attempts by Zhang and Zhang [93] and Andrews and Hemberg [90] provide valuable insights into the performance of methods available at the time. But many more methods have since been proposed and even more comprehensive benchmarking platforms are needed. Some methods, especially those using deep learning, depend strongly on choice of hyperparameters [94]. There, more detailed comparisons that explore parameter spaces would be helpful, extending work like that from Sun et al. [95] comparing dimensionality reduction methods. Such detailed benchmarking would also help to establish when normalization methods derived from explicit count models (e.g., [96, 97]) may be preferable to imputation.

Finally, scalability for large numbers of cells remains an ongoing concern for methods allowing for imputation, as for all high-throughput single-cell methods and software (see “Scaling to higher dimensionalities: more cells, more features, and broader coverage”).

### Challenge II: Defining flexible statistical frameworks for discovering complex differential patterns in gene expression

Beyond simple changes in average gene expression between cell types (or across bulk-collected libraries), scRNA-seq enables a high granularity of changes in expression to be unraveled. Interesting and informative changes in expression patterns can be revealed, as well as cell type-specific changes in cell state across samples (Fig. 6, approach +S). Further understanding of gene expression changes will enable deeper knowledge across a myriad of applications, such as immune responses [98, 99], development [100], and drug responses [101].

#### Status

Currently, the vast majority of differential expression detection methods assume that the groups of cells to be compared are known in advance (e.g., experimental conditions or cell types). However, current analysis pipelines typically rely on clustering or cell type assignment to identify such groups, before downstream differential analysis is performed, without propagating the uncertainty in these assignments or accounting for the double use of data (clustering, differential testing between clusters).

In this context, most methods have focused on comparing average expression between groups [102, 103], but it appears that single cell-specific methods do not uniformly outperform the state-of-the-art bulk methods [104]. Some attention has been given to more general patterns of differential expression (Fig. 3), such as changes in variability that account for mean expression confounding [105], changes in trajectory along pseudotime [106, 107], or more generally, changes in distributions [108]. Furthermore, methods for cross-sample comparisons of gene expression (e.g., cell type-specific changes in cell state across samples; see the “Challenge X: Integration of single-cell data across samples, experiments, and types of measurement”, Fig. 6 and Table 4) are now emerging, such as pseudo-bulk analyses [109–111], where expression is aggregated over multiple cells within each sample, or mixed models, where both within- and between-sample variation is captured [111, 112]. With the expanding capacity of experimental techniques to generate multi-sample scRNA-seq datasets, further general and flexible statistical frameworks will be required to identify complex differential patterns across samples. This will be particularly critical in clinical applications, where cells are collected from multiple patients.

#### Open problems

Accounting for uncertainty in cell type assignment and for double use of data will require, first of all, a systematic study of their impact. Integrative approaches in which clustering and differential testing are simultaneously performed [113] can address both issues. However, integrative methods typically require bespoke implementations, precluding a direct combination between arbitrary clustering and differential testing tools. In such cases, the adaptation of selective inference methods [114] could provide an alternative solution, with an approach based on correcting the selection bias recently proposed [115].

While some methods exist to identify more general patterns of gene expression changes (e.g., variability, distributions), these methods could be further improved by integrating with existing approaches that account for confounding effects such as cell cycle [116] and complex batch effects [117, 118]. Moreover, our capability to dis- cover interesting gene expression patterns will be vastly expanded by connecting with other aspects of single-cell expression dynamics, such as cell type composition, RNA velocity [119], splicing, and allele specificity. This will allow us to fully exploit the granularity contained in single-cell level expression measurements.

In the multi-donor setting, several promising methods have been applied to discover state transitions in high-dimensional cytometry datasets [120–124]. These approaches could be expanded to the higher dimensions and characteristic aspects of scRNA-seq data. Alternatively, there is a large space to explore other general and flexible approaches, such as hierarchical models where information is borrowed across samples or exploring changes in full distributions, while allowing for sample-to-sample variability and subpopulation-specific patterns [111].

### Challenge III: Mapping single cells to a reference atlas

Classifying cells into cell types or states is essential for many secondary analyses. As an example, consider studying and classifying how expression within a cell type varies across different biological conditions (for differential expression analyses, see the “Challenge II: Defining flexible statistical frameworks for discovering complex differential patterns in gene expression” and data integration approach +S in Fig. 6). To put the results of such studies on a map, reliable reference systems with a resolution down to cell states are required—and depending on the research question at hand, even intermediate transition states might be of interest (see “Varying levels of resolution”).

The lack of appropriate, available references has so far implied that only reference-free approaches were conceivable. Here, unsupervised clustering approaches were the predominant option (see data integration approach 1S in Fig. 6). Method development for such unsupervised clustering of cells has already reached a certain level of maturity; for a systematic identification of available techniques, we refer to the respective reviews [125–127].

However, unsupervised approaches involve manual cluster annotation. There are two major caveats: (i) manual annotation is a time-consuming process, which also (ii) puts certain limits to the reproducibility of the results. Cell atlases, as reference systems that systematically capture cell types and states, either tissue specific or across different tissues, remedy this issue (see data integration approach +X+S in Fig. 6). They will need to be able to embed new data points into a stable reference framework that allows for different levels of resolution and will have to eventually capture transitional cell states that fall in between clearly annotated cell clusters (see Fig. 1 for an idea of what cell atlas type reference systems could look like).

#### Status

See Table 3 for a list of cell atlas type references that have recently been published. For human, similar endeavors as for the mouse are under way, with the intention to raise a Human Cell Atlas [5]. Towards this end, initial consortia focus on specific organs, for example, the lung [140].

**Table 3:** Published cell atlases of whole tissues or whole organisms

Organism	Scale of cell atlas	Citation
Nematode (*Caenorhabditis elegans*)	Whole organism at larval stage L2	[128]
Planaria (*Schmidtea mediterranea*)	Whole organism of the adult animal	[129, 130]
Fruit fly (*Drosophila melanogaster*)	Whole organism at embryonic stage	[131]
Zebrafish	Whole organism at embryonic stage	[132, 133]
Frog (*Xenopus tropicalis*)	Whole organism at embryonic stage	[134]
Mouse	Whole adult brain	[135–137]
Mouse	Whole adult organism	[138, 139]

**Table 4:** Approaches for data integration, highlighting their promises and challenges

	Integration	Example MT combination	Example AMs	Promises	Challenges
1S	None	scDNA-seq	Clustering/unsupervised	Discover new subclones, cell types, or cell states	Technical noise ↓; data sparsity ↓
+S	Within 1 MT, within 1 exp, across >1 smps	scRNA-seq	Differential analyses, time series, spatial sampling	Identify effects across sample groups, time, and space	Batch effects ↓; validate cell type assignments ↓
+X+S	Within 1 MT, across >1 exp, across >1 smps	merFISH	Map cells to stable reference (cell atlas)	Accelerate analyses, increase sample size, generalize observations	Standards across experimental centers
+M1C	Across >1 MTs, within 1 exp, within 1 cell	scM&T-seq (scRNA-seq + methylome)	MOFA, DIABLO, MINT	Holistic view of cell state; quantify dependency of MTs	Scaling cell throughput; MT combinations limited; dependency of MTs ↓
+M+C	Across >1 MTs, within 1 exp, across >1 cells, within 1 cell pop	scDNA-seq + scRNA-seq	Cardelino, Clonealign, MATCHER	Use existing datasets (faster than +M1C); flexible experimental design	Validate cell/data matching; test assumptions for integrating data
+all	Across >1 MTs, across >1 exps, across >1 smps, within cells	Hypothetical (any combination)	Hypothetical (map cells to multi-omic HCA, single-cell TCGA)	Holistic view of biological systems	All from approaches +X+S, +M1C, and +M+C

The labeling corresponds to Fig. 6. For each approach, one (combination of) measurement type(s) that is available is given, but more exist and several are discussed in the text. As example analysis methods, actual tool names are given where few tools exist to date; otherwise, broader categories or imaginable methodologies are described

*Abbreviations*: “↓” same challenge also applies to all approaches below, *AM* analysis method, *exp(s)* experiment(s), *HCA* human cell atlas, *MT* measurement type, *smps* samples, *TCGA* The Cancer Genome Atlas

The availability of these reference atlases has led to the active development of methods that make use of them in the context of supervised classification of cell types and states [141–147]. Also, the systematic benchmarking of this dynamic field of tools has begun [148]. A field that can serve as a source of further inspiration is flow/mass cytometry, where several methods already address the classification of high-dimensional cell type data [149–152].

#### Open problems

Cell atlases can still be considered under active development, with several computational challenges still open, in particular referring to the fundamental themes from above [5, 140, 153]. Here, we focus on the mapping of cells or rather their molecular profiles onto stable existing reference atlases to further highlight the importance of these fundamental themes. A computationally and statistically sound method for mapping cells onto atlases for a range of conceivable research questions will need to (i) enable operation at various levels of resolution of interest, and also cover continuous, transient cell states (see “Varying levels of resolution”); (ii) quantify the uncertainty of a particular mapping of cells of unknown type/state (see “Quantifying uncertainty of measurements and analysis results”); (iii) scale to ever more cells and broader coverage of types and states (see “Scaling to higher dimensionalities: more cells, more features, and broader coverage”); and (iv) eventually integrate information generated not only through scRNA-seq experiments, but also through other types of measurements, for example, scDNA-seq or protein expression data (see “Challenge X: Integration of single-cell data across samples, experiments, and types of measurement” for a discussion of data integration, especially approaches +M+C and +all in Fig. 6).

Finally, for further benchmarking of methods that map cells of unknown type or state onto reference atlases (see “Challenge XI: Validating and benchmarking analysis tools for single-cell measurements” for benchmarking in general), atlases of model organisms where full lineages of cells have been determined can form the basis [129, 130, 132, 134, 154]. Importantly, additional information available from lineage tracing of such simpler organisms can provide a cross-check with respect to the transcriptome profile-based classification [134, 155].

### Challenge IV: Generalizing trajectory inference

Several biological processes, such as differentiation, immune response, or cancer expansion, can be described and represented as continuous dynamic changes in cell type/state space using tree, graphical, or probabilistic models. A potential path that a cell can undergo in this continuous space is often referred to as a trajectory ([156] and Fig. 1), and the ordering induced by this path is called pseudotime. Several models have been proposed to describe cell state dynamics starting from transcriptomic data [157]. Trajectory inference is in principle not limited to transcriptomics. Nevertheless, modeling of other measurements, such as proteomic, metabolomic, and epigenomic, or even integrating multiple types of data (see “Challenge X: Integration of single-cell data across samples, experiments, and types of measurement”), is still at its infancy. We believe the study of complex trajectories integrating different data types, especially epigenetics and proteomics information in addition to transcriptomics data, will lead to a more systematic understanding of the processes determining cell fate.

#### Status

Trajectory methods start from a count matrix where genes are rows and cells are columns. First, a feature selection or dimensionality reduction step is used to explore a subspace where distances between cells are more reliable. Next, clustering and minimum spanning trees [156, 158], principal curve or graph fitting [159–161], or random walks and diffusion operations on graphs ([162, 163] among others) are used to infer pseudotime and/or branching trajectories. Alternative probabilistic descriptions can be obtained using optimal transport analysis [164] or approximation of the Fokker-Planck equations [165] or by estimating pseudotime through dimensionality reduction with a Gaussian process latent variable model [166–168].

#### Open problems

Many of the abovementioned methods for trajectory inference can be extended to data obtained with non-transcriptomic assays. For this, the following aspects are crucial. First, it is necessary to define the features to use. For transcriptomic data, the features are well annotated and correspond to expression levels of genes. In contrast, clear-cut features are harder to determine for data such as methylation profiles and chromatin accessibility where signals can refer to individual genomic sites, but also be pooled over sequence features or sequence regions. Second, many of those recent technologies only allow measurement of a quite limited number of cells compared to transcriptomic assays [169–171]. Third, some of those measurements are technically challenging since the input material for each cell is limited (for example, two copies of each chromosome for methylation or chromatin accessibility), giving rise to more sparsity than scRNA-seq. In the latter case, it is necessary to define distance or similarity metrics that take this into account. An alternative approach consists of pooling/combining information from several cells or data imputation (see “Challenge I: Handling sparsity in single-cell RNA sequencing”). For example, imputation has been used for single-cell DNA methylation [172], aggregation over chromatin accessibility peaks from bulk or pseudo-bulk sample [173], and k-mer-based approaches have been proposed [160, 174, 175]. However, so far, no systematic evaluation (see “Challenge XI: Validating and benchmarking analysis tools for single-cell measurements”) of those choices has been performed and it is not clear how many cells are necessary to reliably define those features.

A pressing challenge is to assess how the various trajectory inference methods perform on different data types and importantly to define metrics that are suitable. Also, it is necessary to reason on the ground truth or propose reasonable surrogates (e.g., previous knowledge about developmental processes). Some recent papers explore this idea using scATAC-seq data, an assay to measure chromatin accessibility [160, 174, 176].

Having defined robust methods to reconstruct trajectories from each data type, another future challenge is related to their comparison or alignment. Here, some ideas from recent methods used to align transcriptomic datasets could be extended [118, 177, 178]. A related unsolved problem is that of comparing different trajectories obtained from the same data type but across individuals or conditions, in order to highlight unique and common aspects.

### Challenge V: Finding patterns in spatially resolved measurements

Single-cell spatial transcriptomics or proteomics [179–181] technologies can obtain transcript abundance measurements while retaining spatial coordinates of cells or even transcripts within a tissue (this can be seen as an additional feature space to integrate, see approach +M1C in “Challenge X: Integration of single-cell data across samples, experiments, and types of measurement”, Fig. 6 and Table 4). With such data, the question arises of how spatial information can best be leveraged to find patterns, infer cell types or functions, and classify cells in a given tissue [182].

#### Status

Experimental approaches have been tailored either to systematically extract foci of cells and analyze them with scRNA-seq, or to measure RNA and proteins in situ. Histological sections can be projected in two dimensions while preserving spatial information using sequencing arrays [183]. Whole tissues can be decomposed using the Niche-seq approach [184]: here, a group of cells are specifically labeled with a fluorescent signal, sorted and subjected to scRNA-seq. The Slide-seq approach uses an array of Drop-seq beads with known barcodes to dissolve corresponding slide sites and sequence them with the respective barcodes [185]. Ultimately, one would like to sequence inside a tissue without dissociating the cells and without compromising on the unbiased nature of scRNA-seq. First approaches aiming to implement sequencing by synthesis in situ were proposed by Ke et al. [186] and Lee et al. [187], the latter being referred to as FISSEQ (Fluorescent in situ sequencing). Recently, starMAP [188] was presented. Here, RNA within an intact 3D tissue can be amplified and transferred into a hydrogel. Within the hydrogel, amplified DNA barcodes can be sequenced in situ, in order to distinguish RNA species while retaining spatial coordinates. Instead of performing a direct identification of (parts of) the RNA sequence, fluorescent in situ hybridization (FISH)-based methods require to design probes for targeting RNA species of interest. When multiplexing several rounds of FISH in combination with designed barcodes for each RNA species, it becomes possible to measure hundreds to thousands of RNA species simultaneously. Lubeck et al. [189] have shown a first approach of multiplexed, barcoded FISH to measure tens of RNA species simultaneously, called seqFISH. Later, MERFISH was proposed by Chen et al. [190], which enabled the measurement of hundreds to thousands of transcripts in single cells simultaneously while retaining spatial coordinates [191]. Subsequently, Shah et al. [192] have scaled seqFISH to hundreds of RNA species as well. This year, Eng et al. [193] presented SeqFISH+, which scales the FISH barcoding strategy to 10,000 RNA species by splitting each of 4 barcode locations to be scanned into 20 separate readings to avoid optical signal crowding. The latter can also be an issue when fewer RNA species are measured, in particular at densely populated regions such as the nucleus [190]. To solve such issues at the expense of measuring fewer RNA species, Codeluppi et al. [194] have proposed osmFISH, which uses a single fluorescent probe per RNA species and leverages FISH iterations to measure different species instead of building up a barcode. This leads to a number of recognizable RNA species that is linear in the number of FISH iterations. In addition to the methods that provide in situ measurements of RNA, mass cytometry [195, 196] and multiplexed immunofluorescence [197–199] can be used to quantify the abundance of proteins while preserving subcellular resolution. Finally, the recently described Digital Spatial Profiling [200, DSP; 201] promises to provide both RNA and protein measurements with spatial resolution.

For determining cell types, or clustering cells into groups that conduct a common function, several methods are available [147, 177, 202], but none of these currently use spatial information directly. In contrast, spatial correlation methods have been used to detect the aggregation of proteins [203]. Shah et al. [204] use seqFISH to measure transcript abundance of a set of marker genes while retaining the spatial coordinates of the cells. Cells are clustered by gene expression profiles and then assigned to regions in the brain based on their coordinates in the sample. Recently, Esgärd et al. [205] presented a method to detect spatial differential expression patterns per gene based on marked point processes [206], and Svensson et al. [207] provided a method to perform a spatially resolved differential expression analysis. Here, spatial dependence for each gene is learned by non-parametric regression, enabling the testing of the statistical significance for a gene to be differentially expressed in space.

#### Open problems

The central problem is to consider gene or transcript expression and spatial coordinates of cells, and derive an assignment of cells to classes, functional groups, or cell types. Depending on the studied biological question, it can be useful to constrain assignments with expectations on the homogeneity of the tissue. For example, a set of cells grouped together might be required to appear in one or multiple clusters where little to no other cells are present. Such constraints might depend on the investigated cell types or tissues. For example, in cancer, spatial patterns can occur on multiple scales, ranging from single infiltrating immune cells [208] and minor subclones [209] to larger subclonal structures or the embedding in surrounding normal tissue and the tumor microenvironment [210]. Currently, to the best of our knowledge, there is no method available that would allow the encoding of such prior knowledge while inferring cell types by integrating spatial information with transcript or gene expression. The expected tissue heterogeneity therefore also impacts the desired properties of the assignment method itself. For example, in order to also recognize groups or types of interest that are expected to occur at multiple locations, applicable methods should not strictly rely on co-localization of transcriptional profiles.

Another important aspect when modeling the relation between space and expression is whether uncertainty in the measurements can be propagated to downstream analyses. For example, it is desirable to rely on transcript quantification methods that provide the posterior distribution of transcript expression [102, 211] and propagate this information to the spatial analysis. Since many spatial measurement approaches entail an optical, microscopy-based component, it would be beneficial to extract additional information from these measurements. For example, cell shape and size, as well as the subcellular spatial distribution of transcripts or proteins, could be used to additionally guide the clustering or classification process. Finally, in light of issues with sparsity in single-cell measurements (see “Challenge I: Handling sparsity in single-cell RNA sequencing”), it appears desirable to integrate spatial information into the quantification itself, and, for example, use neighboring cells within the same tissue for imputation or the inference of a posterior distribution of transcript expression.

## Challenges in single-cell genomics

**Fig. 4:**
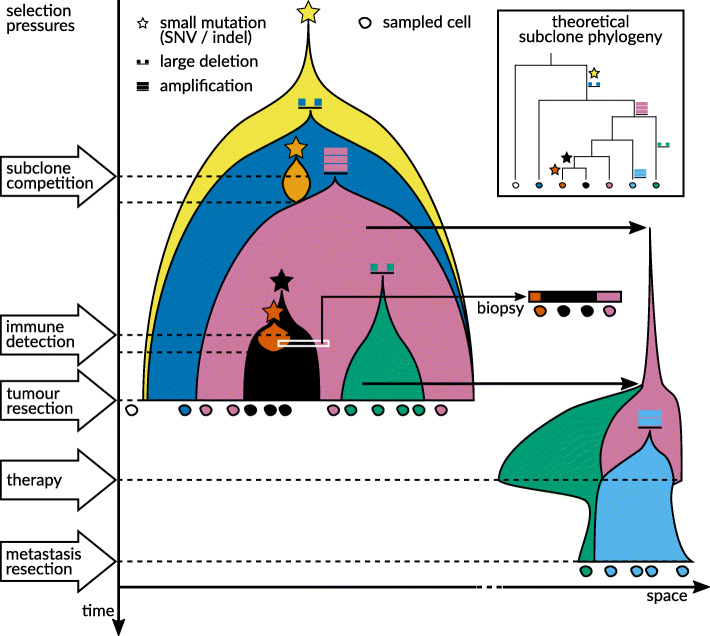
A tumor evolves somatically—from initiation to detection, to resection, and to possible metastasis. New genomic mutations can confer a selective advantage to the resulting new subclone that allows it to outperform other tumor subclones (subclone competition). At the same time, the acting selection pressures can change over time (e.g., due to new subclones arising, the immune system detecting certain subclones, or as a result of therapy). Understanding such selective regimes—and how specific mutations alter a subclone’s susceptibility to changes in selection pressures—will help construct an evolutionary model of tumorigenesis. And it is only within this evolutionary model that more efficient and more patient-specific treatments can be developed. For such a model, unambiguously identifying mutation profiles of subclones via scDNA-seq of resected or biopsied single cells is crucial

With every cell division in an organism, the genome can be altered through mutational events ranging from point mutations, over short insertions and deletions, to large scale copy number variations and complex structural variants. In cancer, the entire repertoire of these genetic events can occur during disease progression (Fig. 4). The resulting tumor cell populations are highly heterogeneous. As tumor heterogeneity can predict patient survival and response to therapy [4, 212], including immunotherapy, quantifying this heterogeneity and understanding its dynamics are crucial for improving diagnosis and therapeutic choices (Fig. 4).

Classic bulk sequencing data of tumor samples taken during surgery are always a mixture of tumor and normal cells (including invading immune cells). This means that disentangling mutational profiles of tumor subclones will always be challenging, which especially holds for rare subclones that could nevertheless be the ones bearing resistance mutation combinations prior to a treatment. Here, the sequencing of single cells holds the exciting promise of directly identifying and characterizing those subclone profiles (Fig. 4).

Ideally, scDNA-seq should provide information about the entire repertoire of distinct events that occurred in the genome of a single cell, such as copy number alterations and genomic rearrangements, together with SNVs and smaller insertion and deletion variants. However, scDNA-seq requires WGA of the DNA extracted from single cells and this amplification introduces errors and biases that present a serious challenge to variant calling [213–216]. It is broadly accepted that different WGA technologies should be used to detect different types of variation. PCR-based approaches [217–220] are best suited for CNV calling, as they achieve a more uniform coverage. But they require thermostable polymerases that withstand the temperature maxima during PCR cycling, and all such polymerases have relatively high error rates. In contrast, MDA-based techniques are the method of choice for SNV calling, as they achieve much lower error rates with the high-fidelity *Φ*29 DNA polymerase [31, 221–225] (in an isothermal reaction, as it would not be stable at common PCR temperature maxima). But MDA suffers from stronger allelic bias in the amplification, possibly because it is more sensitive to DNA input quality [226] and biased priming [227]. The goal must thus be to (i) improve the coverage uniformity of MDA-based methods, (ii) reduce the error rate of the PCR-based methods, or (iii) create new methods that exhibit both a low error rate and a more uniform amplification of alleles. Recent years witnessed intensive research in these directions (see Table 1), promising scalable methodology for scDNA-seq comparable to that already available for scRNA-seq, while at the same time reducing previously limiting errors and biases. While this is not a SCDS challenge, it remains central to continuously and systematically evaluate the whole range of promising WGA methods for the identification of all types of genetic variation from SNVs over smaller insertions and deletions up to copy number variation and structural variants.

### Challenge VI: Dealing with errors and missing data in the identification of variation from single-cell DNA sequencing data

**Fig. 5:**
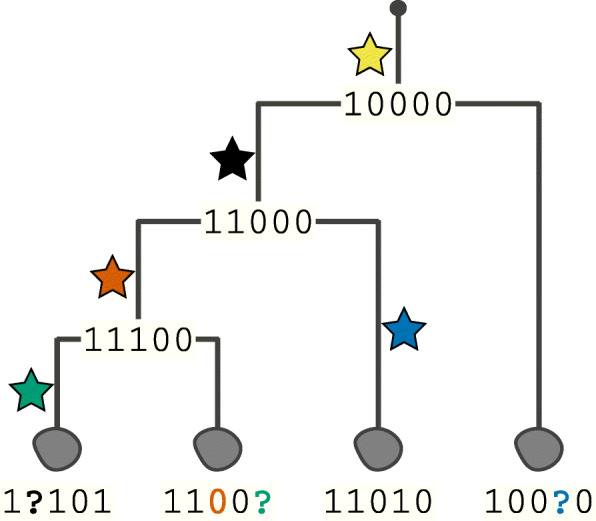
Mutations (colored stars) accumulate in cells during somatic cell divisions and can be used to reconstruct the developmental lineages of individual cells within an organism (leaf nodes of the tree with mutational presence/absence profiles attached). However, insufficient or unbalanced WGA can lead to the dropout of one or both alleles at a genomic site. This can be mitigated by better amplification methods, but also by computational and statistical methods that can account for or impute the missing values

The aim of scDNA-seq usually is to track somatic evolution at the cellular level, that is, at the finest resolution possible relative to the laws of reproduction (cell division, Fig. 5). Examples are identifying heterogeneity and tracking evolution in cancer, as the likely most predominant use case (also see below in “Challenges in single-cell phylogenomics”), but also monitoring the interaction of somatic mutation with developmental and differentiation processes. To track genetic drifts, selective pressures, or other phenomena inherent to the development of cell clones or types (Fig. 4)—but also to stratify cancer patients for the presence of resistant subclones—it is instrumental to genotype and also phase genetic variants in single cells with sufficiently high confidence.

**Fig. 6:**
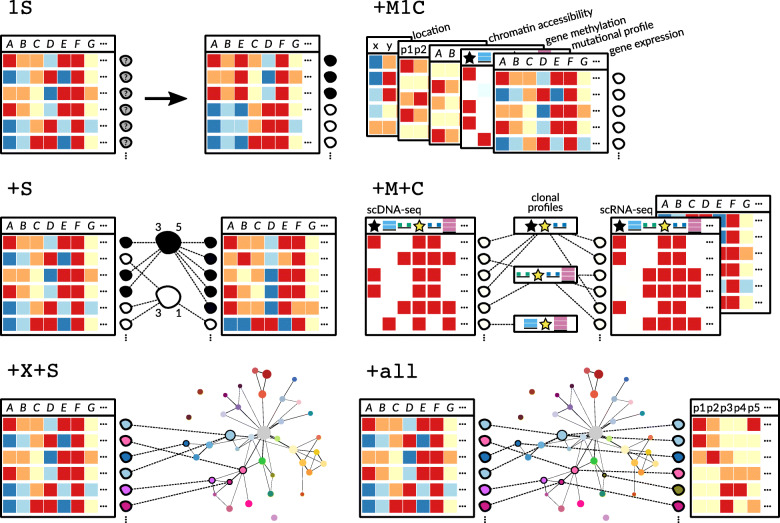
Approaches for integrating single-cell measurement datasets across measurement types, samples, and experiments, as also described in Table 4. 1S: clustering of cells from one sample from one experiment requires no data integration. +S: integration of one measurement type across samples requires the linking of cell populations/clusters. +X+S: integration of one measurement type across experiments conducted in separate laboratories requires stable reference systems like cell atlases (compare Fig. 1). +M1C: integration of multiple measurement types obtained from the same cell highlights the problem of data sparsity of all available measurement types and the dependency of measurement types that needs to be accounted for. +M+C: integration of different measurement types from different cells of the same cell population requires special care in matching cells through meaningful profiles. +all: one possibility for easing data integration across measurement types from separate cells would be to have a stable reference (cell atlas) across multiple measurement types, capturing different cell states, cell populations, and organisms. Effectively, this combines the challenges and promises of the approaches +X+S, +M1C, and +M+C

The major disturbing factor in scDNA-seq data is the WGA process (see above). All methodologies introduce amplification errors (false positive alternative alleles), but more drastic is the effect of amplification bias: the insufficient or complete failure of amplification, which leads to imbalanced proportions or complete lack of variant alleles. Overall, one can distinguish between three cases: an imbalanced proportion of alleles, i.e., loci harboring heterozygous mutations where preferential amplification of one of the two alleles leads to distorted read counts; (ii) allele dropout, i.e., loci harboring heterozygous mutations where only one of the alleles was amplified and sequenced; and (iii) site dropout, which is the complete failure of amplification of both alleles at a site and the resulting lack of any observation of a certain position of the genome. Note that (ii) can be considered an extreme case of (i).

A sound imputation of missing alleles and a sufficiently accurate quantification of uncertainties will yield massive improvements in geno- and haplotyping (phasing) somatic variants. This, in turn, is necessary to substantially improve the identification of subclonal genotypes and the tracking of evolutionary developments. Potential improvements in this area include (i) more explicit accounting for possible scDNA-seq error types, (ii) integrating with different data types with error profiles different from scDNA-seq (e.g., bulk sequencing or RNA sequencing), or (iii) integrating further knowledge of the process of somatic evolution, such as the constraints of phylogenetic relationships among cells, into variant calling models. In this latter context, it is important to realize that somatic evolution is asexual. Thus, no recombination occurs during mitosis, eliminating a major disturbing factor usually encountered when aiming to reconstruct species or population trees from germline mutation profiles.

#### Status

Current single cell-specific SNV callers include Monovar [228], SCcaller [229], and SCAN-SNV [230]. SCcaller detects somatic variants independently for each cell, but accounts for local allelic amplification biases by integrating across neighboring germline single-nucleotide polymorphisms. It exploits the fact that allele dropout affects contiguous regions of the genome large enough to harbor several, and not only one, heterozygous mutation loci. SCAN-SNV works along similar lines, fitting a region-specific allelic balance model to germline heterozygous variants called in a reference bulk sample. Monovar uses an orthogonal approach to variant calling. It does not assume any dependency across sites, but instead handles low and uneven coverage and false positive alternative alleles by integrating the sequencing information across multiple cells. While Monovar merely creates a consensus across cells, integrating across cells is particularly powerful if further knowledge about the dependency structure among cells is incorporated. As pointed out above, due to the lack of recombination, any sample of cells derived from an organism shares an evolutionary history that can be described by a cell lineage tree (see “Challenges in single-cell phylogenomics”). This tree, however, is in general unknown and can in turn only be reconstructed from single-cell mutation profiles. A possible solution is to infer both mutation calls and a cell lineage tree at the same time, an approach taken by a number of existing tools: single-cell Genotyper [231], SciCloneFit [232], and Sci *Φ* [233]. Finally, SSrGE identifies SNVs correlated with gene expression from scRNA-seq data [234].

Some basic approaches to CNV calling from scDNA-seq data are available. These are usually based on hidden Markov models (HMMs) where the hidden variables correspond to copy number states, as, for example, in Aneufinder [235]. Another tool, Ginkgo, provides interactive CNV detection using circular binary segmentation, but is only available as a web-based tool [236]. ScRNA-seq data, which does not suffer from the errors and biases of WGA, can also be used to call CNVs or loss of heterozygosity events: an approach called HoneyBADGER [237] utilizes a probabilistic hidden Markov model, whereas the R package inferCNV simply averages the expression over adjacent genes [238].

#### Open problems

SNV callers for scDNA-seq data have already incorporated amplification error rates and allele dropout in their models. Beyond these rates, the challenge remains to further extend this by directly modeling the amplification process using statistics that would inherently account for both errors and biases, and more accurately quantify the resulting uncertainties (see “Quantifying uncertainty of measurements and analysis results”). This could be achieved by expanding models that accurately quantify uncertainties in related settings [239] and would ultimately even allow reliable control of false discovery rates in the variant discovery and genotyping process. Such expanded models can build on a number of recent studies in this context, for example, on a formalization in a recent preprint [240]. Furthermore, such models could integrate the structure of cell lineage trees with the structure implicit in haplotypes that link alleles. For haplotype phasing, Satas and Raphael [241] recently proposed an approach based on contiguous stretches of amplification bias (similar to SCcaller, see above), whereas others propose read-backed phasing in two recent studies [242, 243]. In addition, the integration with deep bulk sequencing data, as well as with scRNA-seq data, remains unexplored, although it promises to improve the precision of callers without compromising sensitivity.

Identification of short insertions and deletions (indels) is another major challenge to be addressed: we are not aware of any scDNA-seq variant callers with those respective capabilities.

For copy number variation calling, software has previously been published mostly in conjunction with data-driven studies. Here, a systematic analysis of biases in the most common WGA methods for copy number variation calling (including newer methods to come) could further inform method development. The already mentioned approach of leveraging amplification bias for phasing could also be informative [241].

The final challenge is a systematic comparison of tools beyond the respective software publications, which is still lacking for both SNV and CNV callers. This requires systematic benchmarks, which in turn require simulation tools to generate synthetic datasets, as well as real sample-based benchmarking datasets with a reasonably reliable ground truth (see “Challenge XI: Validating and benchmarking analysis tools for single-cell measurements”).

## Challenges in single-cell phylogenomics

Single-cell variation profiles from scDNA-seq, as described above (“Challenge VI: Dealing with errors and missing data in the identification of variation from single-cell DNA sequencing data”), can be used in computational models of somatic evolution, including cancer evolution as an important special case (Fig. 4). For cancer, there is an ongoing, lively discussion about the very nature of evolutionary processes at play, with competing theories such as linear, branching, neutral, and punctuated evolution [244].

Models of cancer evolution may range from a simple binary representation of the presence versus the absence of a particular mutational event (Fig. 5), to elaborate models of the mechanisms and rates of distinct mutational events. There are two main modeling approaches that lend themselves to the analysis of tumor evolution [245]: phylogenetics and population genetics.

Phylogenetics comes with a rich repertoire of computational methods for likelihood-based inference of phylogenetic trees [246]. Traditionally, these methods are used to reconstruct the evolutionary history of a set of distinct species. However, they can also be applied to cancer cells or subclones (Fig. 4). In this setting, tips of the phylogeny (also called leaves or taxa) represent sampled and sequenced cells or subclones, whereas inner nodes (also called ancestral) represent their hypothetical common ancestors. The input for a phylogenetic inference commonly consists of a multiple sequence alignment (MSA) of molecular sequences for the species of interest. For cancer phylogenies, one would concatenate the SNVs (and possibly other variant types) to assemble the input MSA. The key challenge for phylogenetic method development comprises designing sequence evolution models that are (i) biologically realistic and yet (ii) computationally tractable for the increasingly large number of sequenced cells per patient and study.

In population genetics, the tumor is understood as a population of evolving cells (Fig. 4). To date, population genetic theory has been used to model the initiation, progression, and spread of tumors from bulk sequencing data [247–249]. The general mathematical framework behind these models are branching processes [250], for example, in models of the accumulation of driver and passenger mutations [251, 252]. Here, the driver mutations carry a fitness advantage, as might epistatic interactions among them [253]. In contrast, passenger mutations are assumed to be neutral regarding fitness; they merely hitchhike along the fitness advantage of driver mutations they are linked to via their haplotype. The parameters of population genetic models describe inherent features of individual cells that are relevant for the evolution of their populations, for example, fitness and the rates of birth, death, and mutations. Such cell-specific parameters should more naturally apply to and be derived from information gathered by sequencing of individual cells, as opposed to sequencing of bulk tissue samples. Models using these parameters will, for example, be essential in the design of adaptive cancer treatment strategies that aim at managing subclonal tumor composition [254, 255].

### Challenge VII: Scaling phylogenetic models to many cells and many sites

Even if given perfect data, phylogenetic models of tumor evolution would still face the challenge of computational tractability, which is mainly induced by (i) the increasing numbers of cells that are sequenced in cancer studies and (ii) the increasing numbers of sites that can be queried per genome (see “Scaling to higher dimensionalities: more cells, more features, and broader coverage”).

#### Open problems

(i) While adding data from more single cells will help improve the resolution of tumor phylogenies [256, 257], this exacerbates one of the main challenges of phylogenetic inference in general: the immense space of possible tree topologies that grows super-exponentially with the number of taxa—in our case the number of single cells. Phylogenetic inference is NP-hard [258] under most scoring criteria (a scoring criterion takes a given tree and MSA to calculate how well the tree explains the observed data). Calculating the given score on all possible trees to find the tree that best explains the data is computationally not feasible for MSAs containing more than approximately 20 single cells, and thus requires heuristic approaches to explore only promising parts of the tree search space.

(ii) In addition to the growing number of cells (taxa), the breadth of genomic sites and genomic alterations that can be queried per genome also increases. Classical approaches thus need not only scale with the number of single cells queried (see above), but also with the length of the input MSA. Here, previous efforts for parallelization [259, 260] and other optimization efforts [261] exist and can be built upon. The breadth of sequencing data also allows determination of large numbers of invariant sites, which further raises the question of whether including them will change results of phylogenetic inferences in the context of cancer. Excluding invariant sites from the inference has been coined ascertainment bias. For phylogenetic analyses of closely related individuals from a few populations, it has been shown that accounting for ascertainment bias alters branch lengths, but not the resulting tree topologies per se [262].

### Challenge VIII: Integrating multiple types of variation into phylogenetic models

Naturally, downstream analyses—like characterizing intratumoral heterogeneity and inferring its evolutionary history—suffer from the unreliable variant detection in single cells. However, the better the quality of the variant calls becomes, the more important it becomes to model all types of available signal in mathematical models of tumor evolution: from SNVs, over smaller insertions and deletions, to large structural variation and CNVs (Fig. 4). In turn, this should increase the resolution and reliability of the resulting trees.

#### Status

For phylogenetic tree inference from SNVs of single cells, a considerable number of tools exist. The early tools OncoNEM [263] and SCITE [264] use a binary representation of presence or absence of a particular SNV. They account for false negatives, false positives, and missing information in SNV calls, where false negatives are orders of magnitude more likely to occur than false positives. The more recent tool SiFit [265] also uses a binary SNV representation, but infers tumor phylogenies allowing for both noise in the calls and for violations of the infinite sites assumption^2^. Another approach allowing for violations of the infinite sites assumption is the extension of the Dollo parsimony model to allow for *k* losses of a mutation (Dollo-k) [266, 267]. Single-cell genotyper [231], SciCloneFit [232], or Sci *Φ* [233] jointly call mutations in individual cells and estimate the tumor phylogeny of these cells, directly from single-cell raw sequencing data. In a recent work [268], a standard phylogenetic inference tool RAxML-NG [269] has been extended to handle single-cell SNV data. In particular, this implements (i) a 10-state substitution model to represent all possible unphased diploid genotypes and (ii) an explicit error model for allelic dropout and genotyping/amplification errors. Initial experiments showed that—although a 10-state model incorporates more information—it outperformed the ternary model (as used by SiFit) only slightly and only in simulations with very high error rates (10–50%). However, further analysis suggests that benefits of the genotype model become much more pronounced with an increasing number of cells and, in particular, an increasing number of SNVs (preliminary analysis by Kozlov).

While there are no tools yet available to identify insertions and deletions from scDNA-seq (see “Challenge VI: Dealing with errors and missing data in the identification of variation from single-cell DNA sequencing data”), it is only a matter of time until such callers will become available. As they can already be identified from bulk sequencing data, some precious efforts to incorporate indels in addition to substitutions into classical phylogenetic models exist: A decade ago, a simple probabilistic model of indel evolution was proposed [270]. But although some progress has been made since then, such models are less tractable than the respective substitution models [271].

Incorporating CNVs in the reconstruction of tumor phylogeny can be helpful for understanding tumor progressions, as they represent one of the most common mutation types associated to tumor hypermutability [272]. CNVs in single cells were extensively studied in the context of tumor evolution and clonal dynamics [273, 274]. Reconstructing a phylogeny with CNVs is not straightforward. The challenges not only are related to experimental limits, such as the complexity of bulk sequencing data [275] and amplification biases [276], but also involve computational constraints. First of all, the causal mechanisms, such as breakage-fusion-bridge cycles [277] and chromosome missegregation [278], can lead to overlapping copy number events [279]. Secondly, inferring a phylogeny with CNV data requires quantifying biologically motivated transition probabilities for changes in copy numbers. Towards that goal, approaches to calculate the distance between whole copy number profiles [280] are a first step. But for them, a number of challenges remain, with several of the underlying problems known to be NP-hard [280].

Co-occurrence of all of the above variation types further complicates mathematical modeling, as these events are not independent. For example, multiple SNVs that occurred in the process of tumor evolution may disappear at once via a deletion of a large genomic region. In addition, recent analyses revealed recurrence and loss of particular mutational hits at specific sites in the life histories of tumors [281]. This undermines the validity of the so-called infinite sites assumption, commonly made by phylogenetic models.

#### Open problems

For phylogenetic reconstruction from SNVs, we anticipate a shift towards leveraging improvements in input data quality as they are achieved through better amplification methods and SNV callers (see Table 1 and “Challenge VI: Dealing with errors and missing data in the identification of variation from single-cell DNA sequencing data”). For indels, variant callers for scDNA-seq data are anticipated but remain to be developed (see “Challenge VI: Dealing with errors and missing data in the identification of variation from single-cell DNA sequencing data”). Thus, indel modeling efforts for phylogenetic reconstruction from bulk sequencing data should be adapted. For phylogenetic inference from CNVs, the major challenges are (i) determining correct mutational profiles and (ii) computing realistic transition probabilities between those profiles.

The final problem will be to incorporate all of the above phenomena into a holistic model of cancer evolution. However, this will substantially increase the computational cost of reconstructing the evolutionary history of tumor cells. Thus, one needs to carefully determine which phenomena actually do matter (e.g., which parameters even affect the final tree topology) and which features can be measured and called (see “Challenge VI: Dealing with errors and missing data in the identification of variation from single-cell DNA sequencing data”) with sufficient accuracy to actually improve modeling results. As a consequence, one might be able to devise more lightweight models for answering specific questions and invest considerable effort into optimizing novel tools at the algorithmic and technical level (see “Challenge IX: Inferring population genetic parameters of tumor heterogeneity by model integration”).

### Challenge IX: Inferring population genetic parameters of tumor heterogeneity by model integration

Tumor heterogeneity is the result of an evolutionary journey of tumor cell populations through both time and space [209, 212]. Microenvironmental factors like access to the vascular system and infiltration with immune cells differ greatly—for regions within the original tumor as well as between the main tumor and metastases, and across different time points [282]. This imposes different selective pressures on different tumor cells, driving the formation of tumor subclones and thus determining disease progression (including metastatic potential), patient outcome, and susceptibility to treatment ([283, 284] and Fig. 4). However, even the basic questions about the resulting dynamics remain unanswered [285]. For example, it is unclear whether metastatic seeding from the primary tumor occurs early and multiple times in parallel (with metastases diverging genetically from the primary tumor), or whether seeding of metastases occurs late, from a far-developed subclone in the primary tumor (seeding multiple locations with a genotype closer to the late-stage primary tumor). Moreover, it is unknown whether a single cell can seed a metastasis, or whether the joint migration of a set of cells is required. Here, sc-seq can provide invaluable resolution [273].

Although many mathematical models of tumor evolution have been proposed [245, 247, 251, 252, 286, 287], fundamental parameters characterizing the evolutionary processes remain elusive. To quantitatively describe the tumor evolution process and evaluate different possible modes against each other (e.g., modes of metastatic seeding), we would like to estimate fitness values of individual mutations and mutation combinations, as well as rates of mutation, cell birth, and cell death—if possible, on the level of subclones. These parameters determine the underlying fitness landscape of individual cells within their microenvironment, which in turn determines the evolutionary dynamics of cancer progression.

#### Status

Recent technological advances already allow for measuring the arrangement and relationships of tumor cells in space, with cell location basically amounting to a second measurement type requiring data integration within a cell (approach +M1C in “Challenge X: Integration of single-cell data across samples, experiments, and types of measurement”, Fig. 6, and Table 4). While in vivo imaging techniques might also become interesting for obtaining time series data in the future [288], the automated analysis of whole slide immunohistochemistry images [289, 290] seems the most promising in the context of cancer and mutational profiles from scDNA-seq. It is already amenable to single-cell extraction of characterized cells with known spatial context and subsequent scDNA-seq. Using laser capture microdissection [291], hundreds of single cells have recently been isolated from tissue sections and analyzed for copy number variation [292]. For cell and tissue characterization in immunohistochemical images, machine learning models are trained to segment the images and recognize structures within tissues and cells [293–295]: They can, for example, determine the densities and quantities of mitotic nuclei, vascular invasion, and immune cell infiltration on the tissue level, as well as stained biomarkers on the level of the individual cell. These are key parameters of the tumor microenvironment, characterizing the interaction of tumor cells with their environment in space [296, 297], that are key to mathematical models of cancer evolution. Development of reliable classifiers for immunohistochemical images, however, is challenging due to scarcity of training data. Solutions such as active learning can speed up the training process and reduce the workload of annotating pathologists [298].

Classically, mathematical models of tumor population genetics have assumed well mixed populations, ignoring any spatial structure, let alone evolutionary microenvironments. Recently, methods have been extended to account for some spatial structure and have already led to refined predictions of the waiting time to cancer [299] and intratumor heterogeneity [300]. In particular, spatial statistics have been proposed for the quantitative statistical analysis of cancer digital pathology imaging [297], but the idea is applicable to other spatially resolved readouts. Further, a number of methods were proposed to model cell-cell interactions [301, 302] or to predict single-cell expression from microenvironmental features [199, 303].

Regarding temporal resolution, it is already common to sequence tumor material from different time points: biopsies used for diagnosis, resected tumors, lymph nodes and metastases upon surgery, and tumors after relapse. These time points already lend themselves to temporal analyses of clonal dynamics using bulk DNA sequencing data [304], but scDNA-seq is required for a higher resolution of subclonal genotypes. In addition, time resolved measurements and resulting proliferation and death rates promise a higher accuracy in detecting epistatic interactions in cancer genomes than available from previous analyses of bulk sequenced tumor genomes [305–308].

Eventually, population genetic methods and models should be integrated with approaches from phylogenetics, to also leverage the kinship relationships between cells. One prominent example of this recent trend—albeit on bulk data—is the use of the multi-species coalescent model for analyzing MSAs that contain several individuals for several populations [309, 310]. This naturally translates into analyzing tumor subclones as populations of single cells, capturing some of the population structure seen in cancers. Another recent example is a computational model for inference of fitness landscapes of cancer clone populations using scDNA-seq data, SCIFIL [311]. It estimates the maximum likelihood fitness of clone variants by fitting a replicator equation model onto a character-based tumor phylogeny.

For a comprehensive integration, key parameters will need to be quantified with higher resolution. For the detection of positive selection—for example, important in the discussion whether the evolution of tumors is driven by selection or neutral—a number of phylogenetic and population genetic approaches have been proposed in a bulk context. Phylogenetic trees may be used for detecting branches on which positive [312] or diversifying episodic selection [313] is acting.

In this setting, we will have to account for heterotachy (e.g., [314]), that is, we cannot assume a single model of substitution for the entire tree, but have to allow different models to act on distinct branches or subtrees/subclones. Here, anything from a simple model of rate heterogeneity (e.g., [315]) to an empirical mixture model as used for protein evolution [316] could be considered.

#### Open problems

With an increased resolution of scDNA-seq (see “Challenges in single-cell genomics”, Table 1) and more work on the scDNA-seq challenges described in other sections, it will be possible to determine subclone genotypes in more detail. The first challenge will be to integrate this with the spatial location of single cells obtained from other measurements. This will enable determining whether cells from the same subclones are co-located, whether metastases are founded recurrently by the same subclone(s), and whether individual metastases are founded by individual or multiple subclones. Studies utilizing multiple region samples from the same tumor and from distant metastases already paved the way in investigating these questions (e.g., [285]). Still, only single-cell spatial resolution will allow identification of specific individual genotypes in specific locations and drawing precise conclusions.

In addition, it will become possible to determine subclone-specific model parameters and their variability in more detail. For example, rates of proliferation, mutation, and death could be obtained by measuring numbers of mitotic and apoptotic cells per subclone or by integrating subclone abundance profiles across time points. Good estimates of these basic parameters will greatly benefit the detection of positive and negative selection in cancer, and improve the prediction of subclone resistance (and thus expected treatment success) from subclone fitness estimates. The fitness of individual subclones could be calculated from comparing expanded subclones in drug screens under different treatment regimes.

For some of the rates, for example, subclone-specific rates of mutation, the integration of models from population genetics and phylogenetics holds promise and poses a genuine SCDS challenge. But for all of these rates, having better estimates implies follow-up challenges.

One of these resulting challenges will be to detect positive or diversifying selection with greater resolution, building on approaches from the bulk context. Here, tests from the area of “classic” phylogenetics might serve as a starting point for exploring and adapting appropriate methods that will allow to associate positive selection events to branches of the tumor tree or specific evolutionary events. Evolutionary pressures are often quantified by the dN/dS ratio of non-synonymous and synonymous substitutions. In application to tumor cell populations, however, this ratio may not be applicable, as it has been shown to be relatively insensitive when applied to populations within the same species [317]. Other measures have been proposed as better suited for detecting selection within populations based on time series data [318–320] and could potentially be transferred to tumor cell populations.

A particular problem with the detection of positive or diversifying selection is to which extent the above tests will be sensitive to errors in cancer data—the tests are already known to produce high false positive rates in the classic phylogenetic setting when the error rate in the input data is too high [321]. Computationally intense solutions for decreasing the high false positive rate have been proposed [322], but they might not scale to single-cell cancer datasets.

Another resulting problem will be to adapt models for the detection of epistatic interactions to single-cell data. As some of these epistatic interactions can be hard to spot in bulk sequencing data (they may simply disappear because of a low frequency), time-resolved scDNA-seq might be the only way to spot them. If integrated across individuals and cells (see “Challenge X: Integration of single-cell data across samples, experiments, and types of measurement”), it will be possible to identify pairs or even larger combinations of mutations that often occur simultaneously in the same genome, and combinations that rarely or never do. That is, cells affected by negatively selected or synthetic lethal mutations will go extinct in the tumor population, and thus, their genotype with the synthetic lethal mutations occurring together will not be observed. At the same time, cell death can be the result of mere chance, so to detect significant negative pressures, large cohorts of repeated time resolved experiments would have to be performed, resulting in an even larger data integration challenge (see “Challenge X: Integration of single-cell data across samples, experiments, and types of measurement”).

A final step will then be to integrate all these parameters with further information about local microenvironments (such as vascular invasion and immune cell infiltration), to estimate the selection potential of such local factors for or against different subclones.

## Overarching challenges

### Challenge X: Integration of single-cell data across samples, experiments, and types of measurement

Biological processes are complex and dynamic, varying across cells and organisms. To comprehensively analyze such processes, different types of measurements from multiple experiments need to be obtained and integrated. Depending on the actual research question, such experiments can be different time points, tissues, or organisms. For their integration, we need flexible but rigorous statistical and computational frameworks. Figure 6 and Table 4 provide an overview of the promises and challenges of creating such frameworks that we outline here in terms of six approaches of data integration^3^. All of these approaches are affected by the issues that influence single-cell data analysis in general, namely (i) the varying resolution levels that are of interest depending on the research question at hand (see “Varying levels of resolution”), (ii) the uncertainty of any measurements and how to quantify them for and during the analyses (see “Quantifying uncertainty of measurements and analysis results”), and (iii) the scaling of single-cell methodology to more cells and more features measured at once (see “Scaling to higher dimensionalities: more cells, more features, and broader coverage”). All of these further compound the most important challenge in the integration of single-cell data: to link data from different sources in a way that is biologically meaningful and supports the intended analysis. The maps that describe how data from different sources is linked will increase in complexity on increasing amounts of samples, time points, and types of measurements.

In the simplest setup, we obtain one measurement type from multiple cells of a single sample, to identify subpopulations of cells (e.g., subclones or cell types). As any analysis of sc-seq data, it needs to take into account the data’s sparsity (see “Challenge I: Handling sparsity in single-cell RNA sequencing” and “Challenge VI: Dealing with errors and missing data in the identification of variation from single-cell DNA sequencing data”; approach 1S in Fig. 6 and Table 4).

When aiming at identifying patterns of differential expression or characterizing variability across organisms, individuals, or locations, the same measurement type (for example, only scRNA-seq) is taken from multiple samples from different time points, different locations (e.g., different tissues or sites in a tumor), or different organisms (approach +S). Any such combination of samples requires accounting for batch effects among those samples and calls for a validation cell type assignments across samples.

Such batch effects are further aggravated when integrating across multiple experiments, possibly run in different experimental centers with similar but distinct setups (approach +X+S). But standardizing experimental procedures and statistically accounting for batch effects will be well worth the effort wherever this enables a significant increase in sample size, so as to generalize (and statistically corroborate) observations. Nevertheless, even if standards have been successfully established and known batches accounted for, additional validation of, for example, assignments of cells to types and states may be required. Eventually, an increase in generality will support the construction of reference systems, such as a cell atlas, the existence of which can support decisive speed-ups when classifying cells or cell states in subsequent experiments (see “Challenge III: Mapping single cells to a reference atlas”).

Yet another scenario manifests when trying to unravel complexity and coordination of intracellular biological processes, as well as their mutual dependencies, so as to draw a comprehensive picture of a single cell. Here, an optimal setup is to collect several types of measurements from each cell at once; for example, both scDNA-seq and scRNA-seq captured from the same cell, possibly further augmented by measurements of chromatin accessibility, gene methylation, proteins, or metabolites (approach +M1C). The most prominent challenge for this setup is to model inherent dependencies between measurement types wherever phenomena are concurrent (e.g., measuring CNV through scDNA-seq at the same time as obtaining scRNA-seq, with CNV impacting transcription levels).

However, co-measuring different types of quantities in the same cell can be experimentally challenging or even just impossible at this point in time. An exit strategy to this problem is to analyze a population of cells that is homogeneous in terms of some cell type or state, taking different measurement types in different single cells (approach +M+C). After collecting different measurement types in different single cells, one needs to combine the data in a way that is biologically meaningful. An example is to group cells based on commonalities in their genotype profile (Fig. 6), having become evident only after the application of a scDNA-seq experiment. This will require careful validation of the assumptions made when matching cells via such a grouping, possibly including functional validation of group differences.

Finally, the most comprehensive goal will be a holistic view of the complexity of (intra-)cellular circuits, and charting their variability across time, tissues, populations, and organisms (approach +all). Mapping cellular circuits in this comprehensive manner requires integrating complementary and possibly interdependent measurements in single cells and across multiple single cells from diverse samples.

#### Status

For *unsupervised clustering* (approach 1S in Fig. 6 and Table 4), method development is a well-established field. Remaining challenges have already been identified systematically (see [125–127]).

For *integrating datasets across samples in one experiment* (approach +S), a few approaches are available. See for example MNN [118], and the methodologies included in the Seurat package [177, 323, 324]. For the challenges and promises referring to the integration of sc-seq data that vary in terms of spatial and temporal origin, see the discussions in “Challenge V: Finding patterns in spatially resolved measurements” and “Challenge IX: Inferring population genetic parameters of tumor heterogeneity by model integration”.

For *integrating datasets across experiments* (approach +X+S), mapping cells to reference datasets such as the Human Cell Atlas [5] is currently emerging as the most promising strategy. We refer the reader to more particular and detailed discussions in “Challenge III: Mapping single cells to a reference atlas”. While applicable reference systems are not (fully) available, assembling cell type clusters from different experiments is a reasonable strategy, as implemented by several recently published tools [202, 325–332].

*Integrating across multiple measurement types from the same cell* (approach +M1C) has become necessary (and possible) with the advent of experimental protocols that enable the collection of such data [333]. Such protocols combine scDNA-seq and scRNA-seq [333–335]; methylation data and scRNA-seq [336]; all of scRNA-seq, scDNA-seq, methylation, and chromatin accessibility data [41]; or targeted queries on a cell’s genotype, expression (scRNA-seq), and methylation status (sc-GEM [337]). For these single cell-specific approaches, bulk approaches that address the integration of data from different types of experiments have the potential to be adapted to single cell-specific noise characteristics (MOFA [92], DIABLO [338], mixOmics [339], and MINT [340]).

For *integrating across multiple measurement types from separate cells* (approach +M+C), all of which stem from a population of cells that is homogeneous with respect to some selection criterion, technologies such as 10X genomics [171] for scRNA-seq and direct library preparation (DLP [341]) for scDNA-seq establish a scalable experimental basis. The greater analytical challenge is to identify subpopulations that had so far remained invisible, and whose identification is crucial so as to not combine different types of data in mistaken ways. An example for this is the identification of distinct cancer clones from cells sampled from seemingly homogeneous tumor tissue. Here, only performing scDNA-seq experiments can definitively reveal the clonal structure of a tumor. If one wishes to correctly link mutation with transcription profiles, ignoring the clonal structure of a tumor could be misleading. Several analytical methods that address this problem have recently emerged: (i) clonealign [91] assumes a copy number dosage effect on transcription to assign gene expression states to clones, (ii) cardelino [342] aligns clone-specific SNVs in scRNA-seq to those inferred from bulk exome data in order to infer clone-specific expression patterns, and (iii) MATCHER [18] uses manifold alignment to combine scM&T-seq [336] with sc-GEM [337], leveraging the common set of loci. All of these methods are based on biologically meaningful assumptions on how to summarize data measurements across different measurement types and samples, despite their different physical origin.

#### Open problems

Experimental technologies that enable taking multiple measurement types in the same cell (approach +M1C in Fig. 6 and Table 4) are on the rise and will allow to assay more cells at higher fidelity and reduced cost. While this type of data naturally links measurement types within single cells, the SCDS challenge is to account for dependencies among those measurement types for any obtainable combinations of them. As a prominent example, consider how gene expression increases with higher genomic copy number, a phenomenon known as measurement linkage [343], which has not been addressed for different measurement types taken in the same cell. Statistical models for leveraging those measurement type combinations thus pose formidable SCDS challenges.

While progress on the approach +M1C may gradually render approach +M+C obsolete, +M+C will remain the easier—or the only feasible—approach for many measurement type combinations for a while. At the same time, any advances in characterizing dependencies between different measurement types acquired from separate cells (+M+C) provide further ground work for linking them when acquired from the same cell (+M1C). Take the example from above, where copy number profiles will impact gene expression measurements. Here, an approach that accounts for this in +M+C exists (clonealign [91]) and could be extended to +M1C datasets. For approach +M+C, the possibility to integrate data from single cells with data from bulk sequencing of the same cell population also holds promise, for example, by using bulk genotypes for imputation of sites with no sequencing coverage in single cells. Finally, knowing how to link (different) measurement types acquired from different cells is essential for building reference systems across experiments, such as cell atlases (see also approaches +X+S and +all, and “Challenge III: Mapping single cells to a reference atlas”). Thus, exploring further combinations of measurement types and their measurement linkage in +M+C datasets remains as a central SCDS challenge.

No matter which combinations of measurement types become available—the amounts of material underlying most measurements will remain tiny, limited by the amounts within a single cell as well as by a limited number of cells available from a particular cell population. This means that one overarching theme will persist: analyses like training models or mapping quantities on one another will suffer from missing entire views—samples, time points, or measurement types. Thus, integrating data across experiments and different measurement types will further compound the challenge of missing data that we already discussed for non-integrative approaches (see “Challenge I: Handling sparsity in single-cell RNA sequencing” and “Challenge VI: Dealing with errors and missing data in the identification of variation from single-cell DNA sequencing data”).

### Challenge XI: Validating and benchmarking analysis tools for single-cell measurements

With the advances in sc-seq and other single-cell technologies, more and more analysis tools become available for researchers, and even more are being developed and will be published in the near future. Thus, the need for datasets and methods that support systematic benchmarking and evaluation of these tools is becoming increasingly pressing. To be useful and reliable, algorithms and pipelines should be able to pass the following quality control tests: (i) They should produce the expected results (e.g., reconstruct phylogenies, estimate differential expressions, or cluster the data) of high quality and outperform existing methods, if such methods exist. (ii) They should be robust to high levels of sequencing noise and technological biases, including PCR bias, allele dropout, and chimeric signals. In addition, benchmarking should be conducted in a systematic way, following established recommendations [344, 345].

Evaluation of tool performance requires benchmarking datasets with known ground truth. Such data should include cell populations with known genomic compositions and population structures, in other words where frequencies of clones and alleles are known. Currently, such datasets are scarce—with some notable exceptions [346, 347]—because generating them in genuine laboratory settings is time-, labor-, and cost-intensive. Experimental benchmark datasets for evolutionary analysis of single-cell populations are even harder to obtain, as they require follow-up samples with known information about evolutionary trajectories and developmental times. With lack of time-resolved measurements, only anecdotal evidence exists on, for instance, how the accuracy of phylogenetic inferences is affected by data quality. Availability of such gold-standard datasets would benefit single-cell genomics research enormously.

Due to aforementioned difficulties, the most affordable sources of benchmarking and validation data are in silico simulations. Simulations provide ground truth test examples that can be rapidly and cost-effectively generated under different assumptions. However, development of reliable simulation tools requires design and implementation of models that capture the essence of underlying biological processes and technological details of single-cell technologies and high-throughput sequencing platforms, establishing single-cell data simulation as a methodologically involved challenge.

#### Status

Recent studies [104, 111, 148, 157, 348] show that systematic benchmarking of different single-cell analysis methodologies has begun. However, to the best of our knowledge, there is still a shortage of single-cell data simulation tools, for all the possible use cases. Many single-cell data analysis packages include their own ad hoc data simulators [111, 211, 241, 264, 349–353]. However, these simulators are usually not available as separate tools or even as a source code, tailored to specific problems studied in corresponding papers and sometimes not comprehensively documented, thus limiting their utility for the broad research community. Furthermore, since such simulators are used only as auxiliary subroutines inside particular projects and are not published as stand-alone tools, they themselves are usually not guaranteed to be evaluated, and therefore, the accuracy of their reflection of real biological and technological processes can remain unclear. There are few exceptions known to us, including the tools Splatter [354], powsimR [355], and SymSim [356], which provide frameworks for simulation of scRNA-seq data and whose accuracy has been validated by comparison of its results with real data. For single-cell phylogenomics, cancer genome evolution simulators are being designed [357–359].

#### Open problems

Current simulation tools mostly concentrate on differential expression analysis, while comprehensive simulation methods for other important aspects of sc-seq analysis are still to be developed. In particular, to the best of our knowledge, no such tool is available for scDNA-seq data.

With single-cell phylogenomics, one would like to assess the accuracy of methods for phylogenetic inference and subclone identification, or the power of population genetics methods for estimating parameters of interest (e.g., tests for selection and epistatic interactions in cancer, see “Challenge IX: Inferring population genetic parameters of tumor heterogeneity by model integration”). To this end, realistic and comprehensive (w.r.t. the evolutionary phenomena) simulation tools are required.

Another interesting computational problem is the development of tools for validation of simulated sc-seq datasets themselves by their comparison with real data using a comprehensive set of biological parameters. The first such tool for scRNA-seq data is countsimQC [360], but similar tools for scDNA-seq data are needed. Finally, most of the simulators concentrate on modeling of biologically meaningful data, while ignoring or simplifying models for sc-seq errors and artifacts.

Another important challenge in single-cell analysis tool validation is the selection of comprehensive evaluation metrics, which should be used for comparison of different analysis results with each other and with the ground truth. For single-cell data, it is particularly complicated, since many analysis tools deal with heterogeneous clone populations, which possess multiple biological characteristics to be inferred and analyzed. Development of a single measure that captures several of these characteristics is complicated, and in many cases impossible. For example, validation of tools for imputation of cellular and transcriptional heterogeneity should simultaneously evaluate two measures: (i) how close are the reconstructed and true cellular genomic profiles and (ii) how close are reconstructed and true SNV/haplotype frequency distributions. Development of synthetic measures that capture several such characteristics (e.g., based on utilization of earth mover’s distance [361]) is highly important.

When simulating datasets in general, the circularity of simulating and inferring parameters under the same—possibly simplistic—model should be critically assessed, as should potential biases. Thus, further evaluation on empirical datasets for which some ground truth is known will be invaluable. Ideally, all single-cell analysis fields should define a standard set of benchmark datasets that will allow for assessing and comparing methods or come up with a regular data analysis challenge. This approach has been very successful, for example, in protein structure prediction^4^ and metagenomic analyses^5^. A first step in this direction was the recent single-cell transcriptomics DREAM challenge^6^.

Finally, drawing on all the exemplary benchmarking studies mentioned above, it would be immensely beneficial to bring all the required efforts together in a community-supported benchmarking platform: (i) simulating datasets and validating that they capture important characteristics of real data, (ii) curating ground truths for real datasets, and (iii) agreeing on comprehensive evaluation metrics. Ideally, such a benchmarking framework would remain dynamic beyond an initial publication—to allow ongoing comparison of methods as new approaches are proposed and to easily extend it to entirely new fields of method development.

### Supplementary information

**Additional file 1** Review history.

## Abbreviations

CNV: Copy number variation
FISH: Fluorescent in situ hybridization
ICA: Independent component analysis
MALBAC: Multiple annealing and looping-based amplification cycles
MDA: Multiple displacement amplification
MSA: Multiple sequence alignment
NMF: Non-negative matrix factorization
PCA: Principal component analysis
PCR: Polymerase chain reaction
sc-seq: Single-cell sequencing
scDNA-seq: Single-cell DNA sequencing
SCDS: Single-cell data science
scRNA-seq: Single-cell RNA sequencing
SNV: Single nucleotide variation
WGA: Whole genome amplification

## Footnotes

1 Figure 1 was adapted from [14], Fig. 3, provided under Creative Commons Attribution 4.0 International License (http://creativecommons.org/licenses/by/4.0/).

2 The infinite sites assumption posits a genome with an infinite number of sites, thus rendering a repeated mutational hit of the same genomic site along a phylogeny impossible.

3 Graph representation in Fig. 6 approaches +X+S and +all taken from [14], Fig. 3, provided under Creative Commons Attribution 4.0 International License (http://creativecommons.org/licenses/by/4.0/)

4 http://predictioncenter.org/

5 https://data.cami-challenge.org

6 https://www.synapse.org/#!Synapse:syn15665609/wiki/582909
