# Supplementary material for: Eleven grand challenges in single-cell data science
Source: Genome Biol. 2020 Feb 7;21:31. doi: 10.1186/s13059-020-1926-6 (PMC7007675; doi:10.1186/s13059-020-1926-6)
Supplement: Supplementary file 1 — Additional file 1 Review history. [file 13059_2020_1926_MOESM1_ESM.docx]

Review History

**First round of review**

**Reviewer 1**

**Comments to author:**

Lahnemann and colleagues have composed a wide-ranging summary of a series of challenges associated with single-cell methodologies. This manuscript arose from a series of conversations at a conference held at the Lorentz center last year entitled "Single Cell Data Science: Making Sense of Data from Billions of Cells". In this perspective and review, the authors highlight 12 challenges that they foresee requiring attention from the single-cell community at large. These challenges are well organized by theme/category and individually are discussed in a manner that will both educate the general reader as to the specific background associated with that challenge as well as clarify for the reader the details of that challenge. In some cases, the authors suggest potential avenues forwards. In others, they simply state the challenge as they see it. Even in the absence of potential avenues forward, formulating a challenge clearly can be very useful for the field.

Overall, the field of single-cell 'omics is proving to be a rapidly growing field that provides a range of new insights into biological systems, and, as such, this field promises to transform our understanding of biology. Nonetheless, I agree completely with the authors that the unique methodologies and data they produce present substantial challenges that must be met, at least in part, for the exciting promises of single-cell methods to be fulfilled. In this sense, I feel that this perspective/review is timely and will be well received.

I have only a series of minor comments that the authors may wish to consider. Addressing these issues would strengthen the manuscript in my opinion.

1)	In Section 2.2, the authors provide a series of methods in bullet points. It might be easier to follow if these are instead organized in a table. Columns in this table could also be used to draw attention to the differences/similarities of these methods, e.g. a heading like 'Method' could be used to classify the diffusion-based methods versus the graph-based methods.

2)	In Section 3.3 the authors discuss the challenge of generating reference atlases. I completely agree that such atlases will be essential tools in the analysis of cells from tissues that have already been characterized. Here they focus primarily on classifying cells as previously established types, but I wonder if they could comment on how the notion of a reference atlas could be generalized to cell types/states that are not so easily classified into a finite number of categories. For example, in developmental processes cells can meaningfully be between distinct types, or in the context of some tissues, there can be spatial gradients in gene expression such that two cells of the same 'type' nonetheless have distinguishable (and reproducible) gene expression profiles based on their location in the tissue. An approach in which reference atlases are comprised only of a finite number of cell 'type' categories in which newly measured cells will be classified into one of these 'types' would fail to capture such biological information. Perhaps the authors would consider generalizing the notion of reference atlas to include such continuous gene expression profiles or at least discuss briefly how this aspect shapes the 'reference atlas' challenge.

3)	Minor clarification for slide-seq: the method uses drop-seq beads not drop-seq 'drops'

4)	In Challenge V—spatial methods—I find a few important methods are missing. Specifically, Mats Nilsson's in situ sequencing was developed in parallel to FISSEQ and is worth mentioning. Similarly, starMAP is a new technique capable of measuring ~1000 RNAs that is also worth mentioning. Finally, the osmFISH method from the Linnarsson lab, while lower in multiplexing, is a technique that some may find simpler to adopt as compared to MERFISH, starMAP, or seqFISH.

5)	A point of clarification: the original seqFISH paper (2014) cited did not demonstrate hundreds of transcripts but rather only ~10. The first combinatorial sequential single-molecule FISH method to demonstrate hundreds to a thousand transcripts was MERFISH in 2015. seqFISH demonstrated several hundred in Shah et al in 2016.

6)	While the multiplexed immuno methods that measure proteins via scanning mass spec are mentioned—Giesen et al 2014 and Angelo et al 2014—however, it may also be worth mentioning the highly multiplexed immunofluorescence work from Peng Yin's lab, Peter Sorger's lab, and Gary Nolan's lab, (there are also a few others), as these methods provide comparable degrees of multiplexing to the scanning-mass-spec-based methods.

7)	In general, I agree with the author's statement that there are currently no methods that incorporate spatial location during the clustering of cells, and I agree that this is a worthwhile avenue for future development. However, the authors may wish to discuss the notion that there are times where transcriptionally identical cells are actually found in different locations of a tissue. Spatially aware clustering methods risk calling these groups of cells as distinct types, which may not be the best representation of the underlying biology. So there is a risk to spatially aware clustering methods that should be considered as these techniques are developed.

8)	Along these lines, there is additional information provided by the spatial methods that could be very interesting to include in clustering, including cell shape properties such as volume, morphology, etc, or the internal location of the transcriptome or proteome. There is a wealth of data provided by imaging-based methods, and, in my opinion, one of the exciting challenges for the analysis of these data is understanding how best to extract insight from these novel data. Perhaps this point is worth mentioning.

9)	I found the entries in Table 2 a bit confusing. For example, it is not clear if the 'example MTs' column refers to published data of this type from these different methods or, if, the authors simply wish to highlight which methods might require the given approach. Perhaps the authors could clarify this table.

**Reviewer 2**

**Comments to author:**

This paper has some good points to make, but it is very disorganized and poorly written.

Organization:

The paper introduces categories/themes in section 2.

The themes are: (i) quantifying uncertainty, (ii) benchmarking, (i) scaling up to higher dimensional data, (ii) integrating data, and

(iii)determining appropriate levels of resolution. Since this is a list, the labeling (i), (ii), (i), (ii), (iii) is confusing.

The authors then go on to a detailed discussion of (iii), followed by detailed discussions of (i) quantifying uncertainty and (i) scaling. Benchmarking, for example, is discussed again briefly under open questions for some of the challenges, but it is discussed primarily as its own challenge (challenge 12). The same holds true for data integration (Challenge 11). It is not clear why categories/themes are introduced in Section 2 at all.

After these themes are introduced and discussed, challenge categories are discussed (Challenges in sc transcriptomics, genomics and phylogenomics).

Many of the ideas presented within each challenge category are overlapping. For example, Challenge I (handling sparsity) is a challenge in transcriptomics but also phylogenomics. Challenge 12 (benchmarking/validation) underlies each of the challenge categories. Challenge 9 and 11 (integrating data) seem similar. I think the paper would be easier to read if the 12 challenges were stated without trying to categorize: Data integration, sparsity, scaling up, etc...

The paper notes in a few places that it is focused on challenges associated with data analysis methods and so Challenge VI (focused on experimental protocol) seems out of place.

Table 6 is not useful. For example, why are level 0 and levels 3, 4, and 5 not challenged by batch effects? Why are levels 3 and 5 not challenged by

technical noise? Why is only level 3 challenged by sparsity?

Rambling sentences and/or grammatical errors make the paper difficult to read. A few examples from very many are listed below.

First sentence of abstract (have -> has)

The following sentence is poorly written and it's hard to understand the main point on first read. The "by in the meantime" is particularly cumbersome.

"This upswing of high-throughput sc-seq technologies— most importantly in microfluidics techniques and combinatorial indexing strategies [Zilionis et al.,

2017, Vitak et al., 2017, Svensson et al., 2018b, Luo et al., 2019, Gao et al., 2019] — means that tens or hundreds of thousands of cells, instead of just tens or hundreds, are routinely sequenced in one experiment; a development — further fueled by in the meantime low sequencing costs - that has recently even led to a publication on millions of cells in one experiment [Cao et al., 2019a]."

A similar statement could be made about this sentence, and many others.

"Further, a new level of resolution also means another—rapidly growing— dimension in data matrices, thus requiring scalable models and methods for data analysis. While the particular challenges can vary greatly by research goal, tissue analyzed, experimental setup or — last but not least — just by whether DNA or RNA is sequenced, further factoring into various protocols, assaying for example also the epigenome (bisulfite protocols), chromatin accessibility (e.g. ATAC-seq) or protein levels (CITE-seq), the common denominator is that the challenges are all rooted in data science, hence are computational or statistical in nature.

This sentence has three "e.g.'s" in it.

"Such noise can lead to artefactual zero that are either more systematic (e.g. sequence-specific mRNA degradation during cell lysis)

or that occur by chance (e.g. barely expressed transcripts that at the same expression level will sometimes be detected and sometimes not,

due to sampling variation, e.g in the sequencing)."

Page 6: "they they"

**Authors Response**

**Point-by-point responses to the reviewers’ comments:**

Response to Reviewers

*----We thank the reviewers for the thorough review of our manuscript and greatly appreciate all comments. We believe to have addressed all concerns, which has led to a substantially improved manuscript. Please find our detailed response below and all changes to the manuscript highlighted in the PDF with the suffix “_changes_highlighted.pdf”.*

Reviewer #1

Lahnemann and colleagues have composed a wide-ranging summary of a series of challenges associated with single-cell methodologies. This manuscript arose from a series of conversations at a conference held at the Lorentz center last year entitled "Single Cell Data Science: Making Sense of Data from Billions of Cells". In this perspective and review, the authors highlight 12 challenges that they foresee requiring attention from the single-cell community at large. These challenges are well organized by theme/category and individually are discussed in a manner that will both educate the general reader as to the specific background associated with that challenge as well as clarify for the reader the details of that challenge. In some cases, the authors suggest potential avenues forwards. In others, they simply state the challenge as they see it. Even in the absence of potential avenues forward, formulating a challenge clearly can be very useful for the field.

Overall, the field of single-cell 'omics is proving to be a rapidly growing field that provides a range of new insights into biological systems, and, as such, this field promises to transform our understanding of biology. Nonetheless, I agree completely with the authors that the unique methodologies and data they produce present substantial challenges that must be met, at least in part, for the exciting promises of single-cell methods to be fulfilled. In this sense, I feel that this perspective/review is timely and will be well received.

I have only a series of minor comments that the authors may wish to consider. Addressing these issues would strengthen the manuscript in my opinion.

1) In Section 2.2, the authors provide a series of methods in bullet points. It might be easier to follow if these are instead organized in a table. Columns in this table could also be used to draw attention to the differences/similarities of these methods, e.g. a heading like 'Method' could be used to classify the diffusion-based methods versus the graph-based methods.

*----Response: We thank the reviewer for this great suggestion, which we think refers to 3.1 (Challenge I). We have now taken all mentions of methods in this section and moved them to a newly created table. This table follows the classification of the text and provides a short description of each method’s approach, which should emphasize some of the differences and similarities between them. In the process, we have also done further editing in response to Reviewer #2’s general criticism about readability.*

2) In Section 3.3 the authors discuss the challenge of generating reference atlases. I completely agree that such atlases will be essential tools in the analysis of cells from tissues that have already been characterized. Here they focus primarily on classifying cells as previously established types, but I wonder if they could comment on how the notion of a reference atlas could be generalized to cell types/states that are not so easily classified into a finite number of categories. For example, in developmental processes cells can meaningfully be between distinct types, or in the context of some tissues, there can be spatial gradients in gene expression such that two cells of the same 'type' nonetheless have distinguishable (and reproducible) gene expression profiles based on their location in the tissue. An approach in which reference atlases are comprised only of a finite number of cell 'type' categories in which newly measured cells will be classified into one of these

'types' would fail to capture such biological information. Perhaps the authors would consider generalizing the notion of reference atlas to include such continuous gene expression profiles or at least discuss briefly how this aspect shapes the 'reference atlas' challenge.

*----Response: We thank the reviewer for raising this important point. We have further highlighted the importance of transitional intermediate cell states in the context of cell atlases by explicitly mentioning the need to account for those at the end of the introduction to Challenge III (“[Cell atlases] will need to be able to embed new data points into a stable reference framework that allows for different levels of resolution and will have to eventually capture transitional cell states that fall in between clearly annotated cell clusters”). Previously, this was only mentioned in the respective Open problems section.*

3) Minor clarification for slide-seq: the method uses drop-seq beads not drop-seq 'drops'

*----Response: We thank the reviewer for this clarification and have corrected this.*

4) In Challenge V—spatial methods—I find a few important methods are missing. Specifically, Mats Nilsson's in situ sequencing was developed in parallel to FISSEQ and is worth mentioning. Similarly, starMAP is a new technique capable of measuring ~1000 RNAs that is also worth mentioning. Finally, the osmFISH method from the Linnarsson lab, while lower in multiplexing, is a technique that some may find simpler to adopt as compared to MERFISH, starMAP, or seqFISH.

*----Response: We thank a lot for the very useful suggestions and apologize for missing those details initially. The paragraph has now been adapted accordingly.*

5) A point of clarification: the original seqFISH paper (2014) cited did not demonstrate hundreds of transcripts but rather only ~10. The first combinatorial sequential single-molecule FISH method to demonstrate hundreds to a thousand transcripts was MERFISH in 2015. seqFISH demonstrated several hundred in Shah et al in 2016.

*----Response: We thank the reviewer for the clarification, and apologize for being inaccurate. The text has been updated accordingly, also better highlighting the chronological order of the discoveries.*

6) While the multiplexed immuno methods that measure proteins via scanning mass spec are mentioned—Giesen et al 2014 and Angelo et al 2014—however, it may also be worth mentioning the highly multiplexed immunofluorescence work from Peng Yin's lab, Peter Sorger's lab, and Gary Nolan's lab, (there are also a few others), as these methods provide comparable degrees of multiplexing to the scanning-mass-spec-based methods.

*----Response: We have extended the paragraph accordingly and thank for the suggestion.*

7) In general, I agree with the author's statement that there are currently no methods that incorporate spatial location during the clustering of cells, and I agree that this is a worthwhile avenue for future development. However, the authors may wish to discuss the notion that there are times where transcriptionally identical cells are actually found in different locations of a tissue. Spatially aware clustering methods risk calling these groups of cells as distinct types, which may not be the best representation of the underlying biology. So there is a risk to spatially aware clustering methods that should be considered as these techniques are developed.

*----Response: We completely agree with the reviewer. We now try to mention this problem more prominently in the corresponding “open problems” section.*

8) Along these lines, there is additional information provided by the spatial methods that could be very interesting to include in clustering, including cell shape properties such as volume, morphology, etc, or the internal location of the transcriptome or proteome. There is a wealth of data provided by imaging-based methods, and, in my opinion, one of the exciting challenges for the analysis of these data is understanding how best to extract insight from these novel data. Perhaps this point is worth mentioning.

*----Response: Absolutely. We have integrated this excellent idea into the “open problems” section.*

9) I found the entries in Table 2 a bit confusing. For example, it is not clear if the 'example MTs' column refers to published data of this type from these different methods or, if, the authors simply wish to highlight which methods might require the given approach. Perhaps the authors could clarify this table.

*----Response: We apologise for any confusion. To address this, we have cleaned up the table a bit and extended its caption. Specifically, we have restricted ourselves to one example measurement type per integration approach and now explain this choice in the caption. At the same time, we also address a criticism by Reviewer #2 regarding the same table, mostly by removing redundancies in the Challenges column and using more informative labels for the approaches (instead of the plain numbering).*

Reviewer #2

This paper has some good points to make, but it is very disorganized and poorly written.

Organization:

The paper introduces categories/themes in section 2.

The themes are: (i) quantifying uncertainty, (ii) benchmarking, (i) scaling up to higher dimensional data, (ii) integrating data, and (iii) determining appropriate levels of resolution. Since this is a list, the labeling (i), (ii), (i), (ii), (iii) is confusing.

The authors then go on to a detailed discussion of (iii), followed by detailed discussions of (i) quantifying uncertainty and (i) scaling. Benchmarking, for example, is discussed again briefly under open questions for some of the challenges, but it is discussed primarily as its own challenge (challenge 12). The same holds true for data integration (Challenge 11). It is not clear why categories/themes are introduced in Section 2 at all.

After these themes are introduced and discussed, challenge categories are discussed (Challenges in sc transcriptomics, genomics and phylogenomics).

Many of the ideas presented within each challenge category are overlapping. For example, Challenge I (handling sparsity) is a challenge in transcriptomics but also phylogenomics. Challenge 12 (benchmarking/validation) underlies each of the challenge categories. Challenge 9 and 11 (integrating data) seem similar. I think the paper would be easier to read if the 12 challenges were stated without trying to categorize: Data integration, sparsity, scaling up, etc…

*----Response: We thank the reviewer for voicing concerns about confusing numbering and unclear delineations in the categorizations introduced in Section 2. While we agree that Section 2 was not optimal, we think that the categorization will be especially important to readers only interested in a particular field (e.g. single-cell phylogenomics) or data type (transcriptomics).To clear things up, we have done the following:*

*To clearly separate the categorization of challenges from the discussion of recurring themes, we have moved the brief explanation of challenge categories (transcriptomics, genomics, phylogenomics, overarching) to the final paragraphs of the introduction and extended it to further motivate the ways in which we organized the challenges. Thus, Section 2 is only concerned with recurring themes in Single-Cell Data Science.*

*To avoid confusion due to differences in the numbering of the mentioned lists and the respective sections, we have removed this numbering, as it does not add information.*

*In addition, we have further highlighted that we consider the challenges 11 (data integration) and 12 (benchmarking) both as recurring themes that run throughout and as recapitulatory / summarizing challenges in their own right.*

The paper notes in a few places that it is focused on challenges associated with data analysis methods and so Challenge VI (focused on experimental protocol) seems out of place.

*----Response: We thank the reviewer for pointing out that this is, indeed, not a data science challenge. Accordingly, we have removed this challenge. Instead, we provide all essential content in the introduction of the “single cell genomics” category and have moved the summary of recent developments in whole genome amplification methodology into a succinct box for the interested reader.*

Table 6 is not useful. For example, why are level 0 and levels 3, 4, and 5 not challenged by batch effects? Why are levels 3 and 5 not challenged by technical noise? Why is only level 3 challenged by sparsity?

*----Response: We thank the reviewer for pointing out inconsistencies in what we assume was the previous Table 2, now Table 3. At the same time as addressing criticism by Reviewer #1, we have also addressed the points raised here. Namely by making the Challenges column more consistent and cleaning it up (we introduced a symbol for challenges that are introduced once for a simpler integration approach, but also apply for all the more complicated integration approaches further below in the table). In addition, instead of a mere numbering, we now use meaningful labels for the data integration approaches in the Table, the Figure and the text.*

Rambling sentences and/or grammatical errors make the paper difficult to read. A few examples from very many are listed below.

*----Response: We thank the reviewer for emphasizing readability and have addressed this with extensive language editing by four native speakers that are co-authors of the manuscript.*

First sentence of abstract (have -has)

*----Response: We have changed have to has in the first sentence of the abstract.*

The following sentence is poorly written and it's hard to understand the main point on first read. The "by in the meantime" is particularly cumbersome.

"This upswing of high-throughput sc-seq technologies— most importantly in microfluidics techniques and combinatorial indexing strategies [Zilionis et al., 2017, Vitak et al., 2017, Svensson et al., 2018b, Luo et al., 2019, Gao et al., 2019] — means that tens or hundreds of thousands of cells, instead of just tens or hundreds, are routinely sequenced in one experiment; a development — further fueled by in the meantime low sequencing costs - that has recently even led to a publication on millions of cells in one experiment [Cao et al., 2019a]."

*----Response: We thank the reviewer for pointing out this particularly cumbersome sentence and have greatly simplified it. Namely, we have broken it up into 2.5 sentences and have cut a lot of clutter.*

A similar statement could be made about this sentence, and many others.

"Further, a new level of resolution also means another—rapidly growing— dimension in data matrices, thus requiring scalable models and methods for data analysis. While the particular challenges can vary greatly by research goal, tissue analyzed, experimental setup or — last but not least — just by whether DNA or RNA is sequenced, further factoring into various protocols,

assaying for example also the epigenome (bisulfite protocols), chromatin accessibility (e.g. ATAC-seq) or protein levels (CITE-seq), the common denominator is that the challenges are all rooted in data science, hence are computational or statistical in nature.

*----Response: As above, we thank the reviewer for pointing out two more particularly cumbersome sentences. While also editing the other sentences in this paragraph, we have made these particular sentences more readable by cutting superfluous information.*

This sentence has three "e.g.'s" in it.

"Such noise can lead to artefactual zero that are either more systematic (e.g. sequence-specific mRNA degradation during cell lysis) or that occur by chance (e.g. barely expressed transcripts that at the same expression level will sometimes be detected and sometimes not, due to sampling variation, e.g in the sequencing)."

*----Response: We thank the reviewer for pointing out this local cluster of “e.g.”s. We have reduced the second parenthesis to one “e.g.” and have hopefully made this example more readable in the process.*

Page 6: "they they"

*----Response: We thank the reviewer for pointing out this typo and have fixed it.*

**Second round of review**

**Reviewer 2**

The authors have submitted a substantial revision and have responded to my comments.
